# Exploring Acceptability Drivers of Oral Antibiotics in Children: Findings from an International Observational Study

**DOI:** 10.3390/pharmaceutics13101721

**Published:** 2021-10-18

**Authors:** Thibault Vallet, Yahya Bensouda, Jumpei Saito, Liv Mathiesen, Varsha Pokharkar, Viviane Klingmann, Matthew Peak, Omar Elhamdaoui, Akimasa Yamatani, Ivana Ivanovic, Manjusha Sajith, Juliane Münch, Louise Bracken, Jennifer Claire Duncan, Smita Salunke, Siri Wang, Fabrice Ruiz

**Affiliations:** 1 ClinSearch, 92240 Malakoff, France; thibault.vallet@clinsearch.net; 2 Faculty of Pharmacy and Medicine, Mohammed V University in Rabat, Rabat 10170, Morocco; y.bensouda@um5s.net.ma (Y.B.); omar.elhamdaoui@um5s.net.ma (O.E.); 3 Specialties Hospital, University Medical Centre Ibn Sina (CHIS), Rabat 10170, Morocco; 4 National Center for Child Health and Development, Tokyo 157-8535, Japan; saito-jn@ncchd.go.jp (J.S.); yamatani-a@ncchd.go.jp (A.Y.); 5 Department of Pharmacy, Section for Pharmacology and Pharmaceutical Biosciences, University of Oslo, 0316 Oslo, Norway; livmathi@farmasi.uio.no (L.M.); ivana.ivanovic@apotek1.no (I.I.); 6 Poona College of Pharmacy, Bharati Vidyapeeth Deemed University, Pune 411038, India; varsha.pokharkar@bharatividyapeeth.edu (V.P.); manjusha.sajith@bharatividyapeeth.edu (M.S.); 7 Department of General Paediatrics, Neonatology and Paediatric Cardiology, University Hospital Düsseldorf, 40225 Düsseldorf, Germany; viviane.klingmann@med.uni-duesseldorf.de (V.K.); juliane.muench@med.uni-duesseldorf.de (J.M.); 8 Paediatric Medicines Research Unit, Institute in the Park, Alder Hey Children’s NHS Foundation Trust, Eaton Road, Liverpool L12 2AP, UK; matthew.peak@alderhey.nhs.uk (M.P.); louise.bracken@alderhey.nhs.uk (L.B.); jennifer.duncan@alderhey.nhs.uk (J.C.D.); 9 Department of Pharmaceutics, University College London School of Pharmacy, London WC1N 1AX, UK; s.salunke@ucl.ac.uk; 10 Norwegian Medicines Agency, 0213 Oslo, Norway; siri.wang@legemiddelverket.no

**Keywords:** acceptability, antibiotic, formulation, pediatric, children, ClinSearch acceptability score test (CAST)

## Abstract

Antibiotics are among the most commonly prescribed drugs in children. Adherence to the treatment with these drugs is of the utmost importance to prevent the emergence of resistant bacteria, a global health threat. In children, medicine acceptability is likely to have a significant impact on compliance. Herein we used a multivariate approach, considering simultaneously the many aspects of acceptability to explore the drivers of oral antibiotic acceptability in children under twelve, especially in toddlers and in preschoolers. Based on 628 real-life observer reports of the intake of 133 distinct medicines, the acceptability reference framework highlighted the influence of many factors such as age and sex of patients, previous exposure to treatment, place of administration, administration device, flavor agent in excipients and active pharmaceutical ingredient. These findings from an international observational study emphasize the multidimensional nature of acceptability. Therefore, it is crucial to consider all these different aspects for assessing this multi-faceted concept and designing or prescribing a medicine in order to reach adequate acceptability in the target population.

## 1. Introduction

Antimicrobial resistance is one of the biggest threats to global health and optimizing the use of antimicrobial medicines is one of the five strategic objectives of the ‘WHO Global action plan on antimicrobial resistance’ [1,2]. The unnecessary and inappropriate prescription of antibiotics as well as poor patient adherence are significant contributing factors in the development of antibiotic resistance [3,4,5,6]. Despite the importance of completing the prescribed course of antibiotic therapy as well as promoting the rational use of antimicrobial drugs, rapid spread of bacterial resistance and poor patient adherence continue to be a global challenge.

The rate of antibiotic prescription is significantly higher in children than in adults. Young children have been identified as the highest users of antibiotics, and the use of antibiotics has been repeatedly shown to be much higher among younger children than among older children [7,8,9,10]. Hence, the risk of antibacterial resistance is high in children. The majority of antibiotics are orally administered and many childhood infections are commonly treated with those medicines [11,12,13]. The study by Li et al. showed that less than a quarter of child-appropriate oral antibiotic formulation sales in 2015 globally were in the form of dispersible tablets. Moreover, two thirds were sold as liquid formulations [14]. There is disparity of acceptability among the different antibiotics prescribed for children even for the same drug [15].

Acceptability of medicinal products has emerged as a major factor in adherence [16]. The European Medicines Agency (EMA) has defined patient acceptability as, “The overall ability and willingness of the patient to use and its caregiver to administer the medicine as intended”. Many referenced studies have highlighted the impact on user’s perception of such formulation differences among distinct antibiotic products, especially variations in terms of flavors [15,17,18,19,20,21,22]. However, the available information is still fragmented. Although palatability appeared to be a key aspect of antibiotic acceptability [23], this multifaceted concept is driven by many other parameters. Indeed, multiple features of the medication itself (e.g., swallowability, usability), characteristics of antibiotic course (e.g., number of daily doses, treatment duration), characteristics of the patient (e.g., age, sex, health-status, culture), attitude of patients/carers towards antibiotics, characteristics of medical condition, as well as the specific context of use (e.g., place, time of administration) may affect acceptability [16]. In this class of medicine, many reference products (princeps) have generics. Although the lower acquisition cost permits the use of those generic products that are bioequivalent to the princeps, formulation differences (e.g., tablet size, flavors, sweeteners, coloring agents) might affect medicine acceptability and consequently, patient adherence [24]. Knowledge of the acceptability drivers of oral antibiotics will help strengthen the prescribing/dispensing of appropriate formulations, ensuring adequate patient adherence and consequently, promoting rational use of antibiotics in pediatrics.

Herein we used a multivariate approach [25,26,27,28,29,30] mining a large set of international real-life data to explore acceptability drivers of oral antibiotics in children.

## 2. Materials and Methods

### 2.1. Study Design, Objective and Setting

An international, multicenter, prospective, observational study aiming to investigate the drivers of medicine acceptability in pediatrics was started in May 2015. For this purpose, a large set of real-life observer reports for the use of various medicinal products in a wide spectrum of patients under 18 years old were gathered. Data were collected at home and at hospital in France (May 2015–December 2020); at hospital in Morocco (November 2018–February 2020), the United-Kingdom (June 2019–April 2021), Japan (June 2019–July 2021), India (August 2019–January 2021) and Germany (August 2020–June 2021); at home in Norway (May 2018–June 2021), Poland (July 2020) and Peru (May 2021–June 2021). The ClinSearch Acceptability Score Test (CAST), a standardized and validated tool, was used to explore medicine acceptability drivers [25,26,27,28,29,30]. The published methodology is briefly described in the following subsections.

This article focuses exclusively on findings related to the prescription and administration of oral antibiotics in children aged 0 to 12 years, with a particular focus on toddlers and preschoolers (2–5 years).

### 2.2. Data Collection

**Figure 1:**
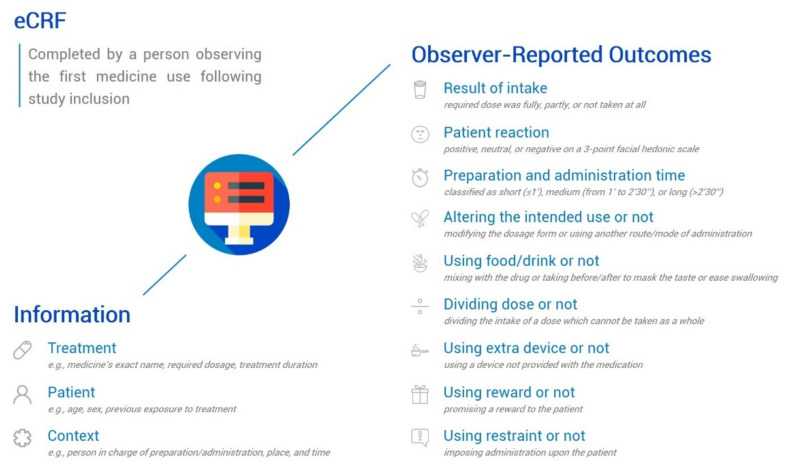
Data collection questionnaire.

Once a patient enrolled into the study, an electronic case report form (eCRF) accessible on a web-based platform was completed by a person observing the first medicine use following study inclusion. The eCRF focused only on the first medicine intake, to avoid bias caused by prior administration of other medicines if the patient was on more than one medicine. The observer was a carer (e.g., family member, other adult helper) for outpatients taking their medication at home, or a healthcare professional (e.g., researcher, doctor, nurse, monitor) for inpatients treated at hospital. The observer reported objective measures including events/behaviors that were observed during the medicine administration (Figure 1). Excluding inferences about the patient’s subjective experience made by informants, observations were suitable to collect reliable information in children. In addition, the observer reported information on the patient, the treatment, and the context of use (Figure 1).

Further information on the medicine which may influence acceptability was collected from the summary of product characteristics (e.g., excipients, administration device) by members of the site study team.

### 2.3. Data Analysis

Medicine acceptability was scored using the acceptability reference framework: a three-dimensional map (3D-map) juxtaposing two distinct acceptability profiles—”Positively accepted” and “Negatively accepted”—materialized by the green and the red areas on the map, respectively.

The published analytical procedure is succinctly described hereafter [25,26,27,28,29,30]. Unsupervised methods were used to visualize the major information from the large set of 3130 standardized evaluations collected in the entire acceptability study. A multiple correspondence analysis summarized in a low-dimensional Euclidean space the main underlying structures of the dataset—each of the 3130 rows corresponded to one evaluation, each of the nine columns represented one constituting observational variable (e.g., the result of the intake), with an observed measure (e.g., the required dose was fully taken) being entered into each corresponding cell. The three dimensions forming the map revealed the associations and dissociations of observed measures that contributed the most to explaining variability observed in the data. Proximities between elements on the 3D-map reflected similarities. Subsequently, hierarchical clustering on principal components and *k*-means consolidation partitioned the set of evaluations into clusters according to their Euclidean distances. The clusters characterized by the observed measures significantly over-represented into each of them, defined acceptability profiles. Therefore, all the evaluations of a particular medicinal product taken by individual patients in a specific context were positioned into the reference framework according to the nine constituting variables. Then, the acceptability reference framework provided comprehensive acceptability scores, partitioning the evaluations according to the explanatory variables (e.g., the age of the patient, the pharmaceutical form of the medicine) as described hereafter.

Among all the evaluations, those focusing on oral antibiotics in children under twelve were selected. The barycenter of this subset of evaluations defined the position on the acceptability map of oral antibiotics considered as a whole in children. To be classified as positively accepted the barycenter, along with the entire 90% confidence ellipsis surrounding it, should reside in the green area of the map. In order to explore acceptability drivers, this subset of evaluations of oral antibiotics was successively partitioned into subgroups according to the patient characteristics, the product characteristics and the context of use. Acceptability scoring was similarly performed for all subgroups of interest. A minimum of 30 evaluations was required to obtain a reliable score with satisfactory precision, and different acceptability scores were significantly different if confidence ellipses did not overlap on the map.

For all the subgroups of interest that were compared to investigate a specific aspect, the significance of the differences observed in terms of setting (place of administration, country), patient characteristics (age group, sex, treatment exposure) and product characteristics (active pharmaceutical ingredient (API), pharmaceutical form category, administration device provided and flavor for oral liquids) was assessed in order to identify any confounding factors. Pearson’s chi-squared test was used. Alternatively, Fisher’s exact test was used when there were few observations for individual cells of the contingency table (less than an expectation of 5 for 20%) or null expectation.

Data analysis was performed using R version 1.0.136© (RStudio Team (2016). RStudio: Integrated Development for R. RStudio, Inc., Boston, MA, USA). The R packages FactoMineR [31] and missMDA [32] were used to perform multivariate analysis—mapping and clustering—and to handle missing data, respectively.

## 3. Results

### 3.1. Patients and Medicines

Among all the 3130 evaluations that gave rise to the acceptability reference framework, there were 628 evaluations of oral antibiotics in patients under 12 years of age. The mean age of the patients was 3.7 years (SD = 3) and 47% were girls. There were 133 distinct medicinal products assessed for 22 APIs and 16 distinct dosage forms. Penicillins (40%) and cephalosporins (26%) were the most common categories prescribed followed by sulfonamides (14%) and macrolides (12%). The most frequently prescribed antibiotic agent was amoxicillin, alone or in combination with clavulanic acid. Seventy-eight percent of the evaluations were associated on reconstituted oral liquid preparations such as powders, which must be dissolved or dispersed in water prior to administration. Fifteen percent of evaluations were on oral liquids ready to use and 7% on solid oral dosage forms. Table 1 presents the characteristics of the patients and products, stratified by patient age.

### 3.2. Acceptability Drivers

**Figure 2:**
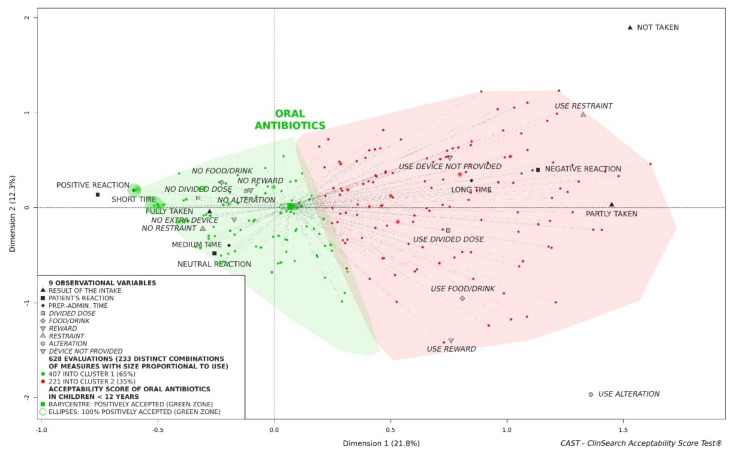
Acceptability scoring of oral antibiotics in children under twelve years of age.

Oral antibiotics in children under 12 years of age considered as a whole were positioned on the acceptability reference framework at the barycenter of the 628 evaluations (Figure 2). As expected, many distinct combinations of observed measures had been used (*n* = 233) reflecting important heterogeneity of evaluations due to the wide variety of products and users included in the study. The reference framework allows exploration of acceptability differences in subgroups of patients/products.

#### 3.2.1. Influence of Age of Children on Acceptability of Oral Antibiotics

**Figure 3:**
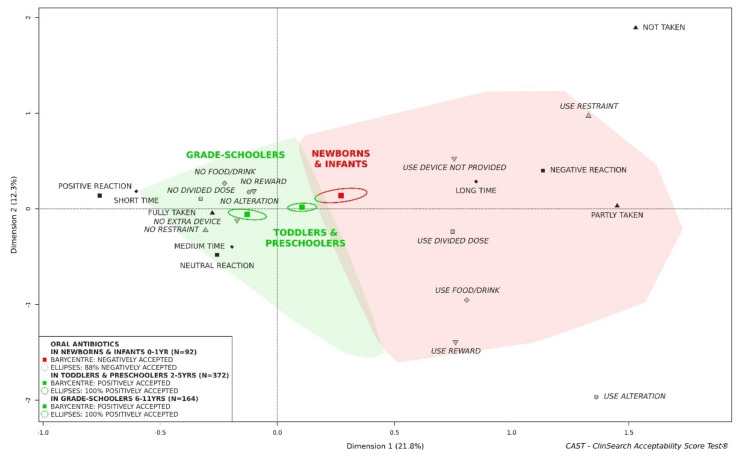
Acceptability of oral antibiotics in newborns and infants, toddlers and preschoolers, and grade-schoolers.

We initially focused on a key factor influencing the acceptability in pediatrics: the age of children. Figure 3 highlights the influence of age on acceptability of oral antibiotics. Oral antibiotics could not be considered as acceptable in newborns and infants (0–1 year), while they appeared to be positively accepted in older children. Differences in acceptability score reflected significant differences for seven of the nine observational variables describing the many aspects of acceptability: the required dose of oral liquid antibiotics was fully taken for 96% of evaluations in grade-schoolers (6–11 years), 81% in toddlers and preschoolers (2–5 years), and 77% in newborns and infants (0–1 year) (χ^2^: *p* < 0.001); the patient reaction was negative for 23% of evaluations in grade-schoolers, 35% in toddlers and preschoolers, and 43% in newborns and infants (χ^2^: *p* < 0.001); the preparation and administration time was longer than 2 min and 30 s for 34% of evaluations in grade-schoolers, 40% in toddlers and preschoolers, and 59% in newborns and infants (χ^2^: *p* < 0.001); the intake of the required dose was divided for 19% of evaluations in grade-schoolers, 37% in toddlers and preschoolers, and 52% in newborns and infants (χ^2^: *p* < 0.001); a reward was used for 14% of evaluations in grade-schoolers, 19% in toddlers and preschoolers, and only 2% in newborns and infants (χ^2^: *p* < 0.001); the patient had to be made to take the medicine for 7% of evaluations in grade-schoolers, 22% in toddlers and preschoolers, and 38% in newborns and infants (χ^2^: *p* < 0.001); a device not provided with the medicine was used for 16% of evaluations in grade-schoolers, 27% in toddlers and preschoolers, and 29% in newborns and infants (χ^2^: *p* = 0.029). There was no significant difference observed for using food/drink to mask the drug taste or ease swallowing (19% for children 0–1, 22% for children 2–5, and 28% for children 6–11) and alterations with regard to the intended form of use (4% for children 0–1, 5% for children 2–5, and 6% for children 6–11). Table 1 presents the differences in terms of patients, products and settings between the three age groups. There were significant differences in terms of treatment exposure, place of administration, countries, APIs and pharmaceutical form categories.

Toddlers and preschoolers—the middle group on the acceptability map—represented the largest group of evaluations (*n* = 372) facilitating subgroups exploration.

#### 3.2.2. Influence of Dosage Form on Acceptability of Oral Antibiotics

Solid oral dosage forms (SODFs) tended to be accepted in grade-schoolers—the barycenter of the 27 evaluations (16.5% of the 164 evaluations), along with the entire confidence ellipsis, was positioned in the green area of the 3D-map—while this tended not to be the case for toddlers and preschoolers—the barycenter of the 15 evaluations (4% of the 372 evaluations), along with 76% of the confidence ellipsis, was positioned in the red area of the 3D-map. Toddlers and preschoolers were for the most part treated with oral liquids: 96% of the evaluations in this study. Although the barycenter of these evaluations, along with the confidence ellipses surrounding it, was positioned in the positively accepted area, acceptability changes were observed based on product, patient and context features.

In toddlers and preschoolers, there was no difference in acceptability score interpretation between the reconstituted oral liquids (*n* = 306) and the oral liquid ready-to-use (*n* = 51): both were positively accepted and ellipses overlapped. There was a similar interpretation in grade-schoolers although oral liquid ready-to-use medicines were significantly further from the red area than reconstituted oral liquids. Both oral liquid forms were not positively accepted in newborns and infants.

#### 3.2.3. Influence of Administration Device Provided with the Medicine on Acceptability of Oral Liquid Formulations of Antibiotics

**Figure 4:**
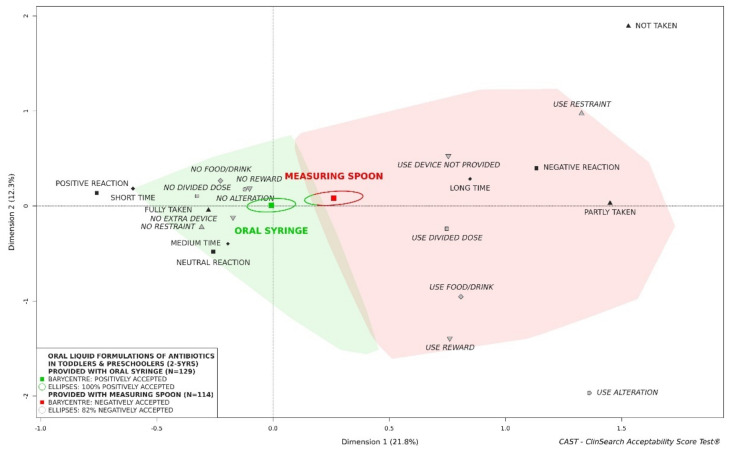
Acceptability of oral liquid formulations of antibiotics in toddlers and preschoolers depending on administration device.

Regardless of the forms, an effect of the administration device on acceptability was observed in toddlers and preschoolers: oral liquid preparations of antibiotics provided with an oral syringe were significantly better accepted than those supplied with a measuring spoon (Figure 4). There were differences between the two subgroups of evaluations in terms of place of administration, countries, APIs, pharmaceutical form categories and flavor composition (Table S1). In terms of administration devices, there was no significant difference in grade-schoolers—oral antibiotics supplied with both administration devices were positively accepted (Figure S1)—whereas in newborns and infants, oral antibiotics tended to not be accepted regardless of the provided administration device (Figure S2).

#### 3.2.4. Influence of Administration Setting on Acceptability of Oral Liquid Formulations of Antibiotics

**Figure 5:**
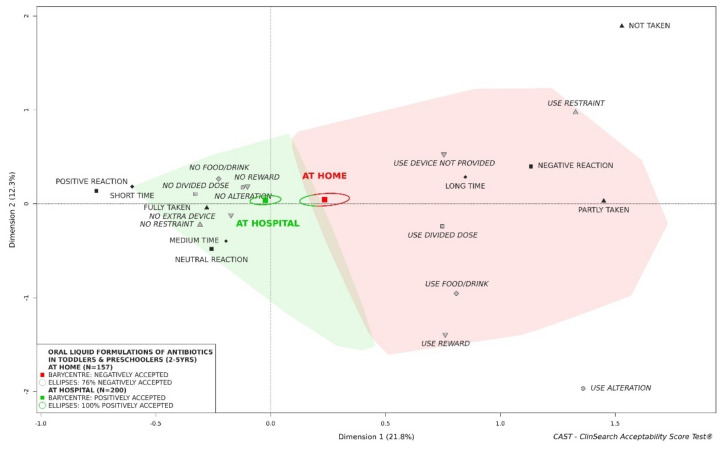
Acceptability of oral liquid formulations of antibiotics in toddlers and preschoolers depending on place of administration.

An effect of the administration setting was observed in the toddler and preschooler group: antibiotics as oral liquids appeared to be better accepted in hospital compared to at home (Figure 5). In terms of recruitment, there were significant differences between both subgroups for countries, APIs, pharmaceutical form categories, the administration device provided and flavor composition (Table S2). For grade-schoolers, the hospital subgroup was similarly located further from the “negatively accepted” profile materialized in red on the right of the map, than the home subgroup (Figure S3). In newborns and infants, the reverse occurred even if ellipses largely overlapped: the barycenter of the 61 evaluations from hospital, along with 94% confidence ellipsis surrounding it, was located in the red area of the map, while 29% of the confidence ellipsis were located in the green area for home (Figure S4).

#### 3.2.5. Influence of Previous Exposure to Treatment on Acceptability of Oral Liquid Formulations of Antibiotics

**Figure 6:**
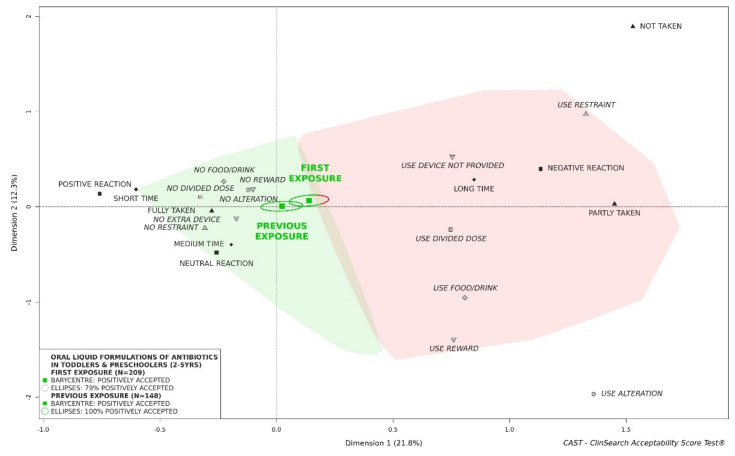
Acceptability of oral liquid formulations of antibiotics in toddlers and preschoolers depending on exposure to treatment.

Regarding patient features, we observed an effect of exposure to treatment on acceptability. Considered as a whole, the medicines which children had been exposed to previously were classified as accepted, while it was not the case when it was the first intake of the medicinal product (Figure 6). However, the difference was not significant as the ellipses overlapped. There were differences between the two subgroups of evaluations in terms of countries, APIs, administration device provided and flavor composition (Table S3). Furthermore, there was no significant difference in grade-schoolers—”previous exposure” and “first exposure” were positively accepted (Figure S5)—in contrast to newborns and infants, where both groups tended to be not accepted (Figure S6).

#### 3.2.6. Influence of Sex of Children on Acceptability of Oral Liquid Formulations of Antibiotics

**Figure 7:**
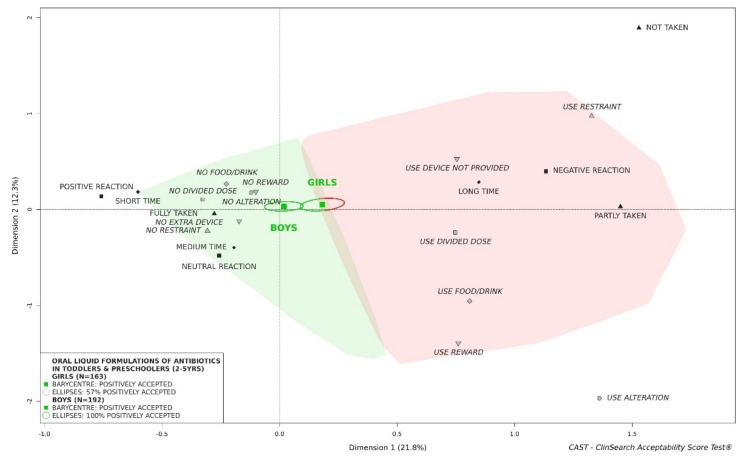
Acceptability of oral liquid formulations of antibiotics in toddlers and preschoolers depending on sex of patients.

The findings indicate that oral liquid preparations of antibiotics were positively accepted by boys, but not by girls: 43% of confidence ellipses were located in the red area of the 3D-map-in toddlers and preschoolers (Figure 7). There was no difference in terms of patient characteristics between the subgroups, but there were differences in terms of product features such as APIs, pharmaceutical form categories and flavor composition (Table S4). In newborns and infants, although ellipses overlapped and barycenters were in the red area, 50% of the confidence ellipsis was located in the green area for boys, while the confidence ellipsis were fully located in the red area of the 3D-map for girls (Figure S7). In grade-schoolers, there was no difference observed: antibiotics as oral liquids were similarly positively accepted by both boys and girls (Figure S8). This general observation for oral liquid antibiotics considered as a whole was not valid in any case for any drug, e.g., the position of amoxicillin and clavulanic acid (co-amoxiclav)—the antibiotic most assessed in the study—on the 3D-map was quite similar for both sexes: 78% of confidence ellipses in the “positively” accepted profile for boys and 75% for girls (Figure S9). There was no significant difference in characteristics between the two subgroups of evaluations for co-amoxiclav (Table S5).

#### 3.2.7. Influence of Flavor on Acceptability of Oral Liquid Formulations of Antibiotics

**Figure 8:**
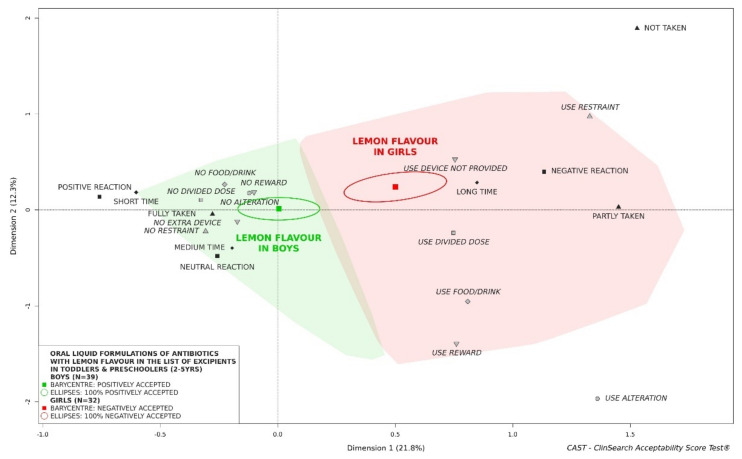
Acceptability of oral liquid formulations of antibiotics with lemon flavor in the list of excipients in toddlers and preschoolers depending on sex of patients.

Occasionally, certain aromas such as lemon seem to have contrasting effects on girls and boys: “positively accepted” by boys and “negatively accepted” by girls (Figure 8). There was no significant difference in terms of age groups, exposure to treatment, place of administration, countries, APIs, pharmaceutical form categories and provided administration device between the two subgroups of evaluations with lemon aroma in the list of excipients (Table S6). There was only a slight difference in term of lemon association in flavor composition. Other aromas such as strawberry were positively accepted by both girls and boys (Figure S10, Table S7), while banana tended (*n* < 30) to be poorly accepted regardless of sex (Figure S11, Table S8). There was only a significant difference of country between the two subgroups of evaluations for strawberry (Table S7).

Considered as a whole, oral liquid formulations of antibiotics with strawberry aroma in the list of excipients were classified as accepted. However, they did not seem to be similarly accepted depending on APIs: cefixime was the furthest from the “negatively accepted” profile, josamycin was also fully located in the green area and co-amoxiclav (amoxicillin/clavulanic acid) had 22% of confidence ellipses in the red area (Figure S12). However, considering the insufficient number of patients for banana flavor (*n* < 30) we could only describe acceptability tendency. Furthermore, there were significant differences between the three subgroups of evaluations in terms of place of administration, countries, and strawberry association in flavor composition (Table S9).

#### 3.2.8. Influence of Active Pharmaceutical Ingredient on Acceptability of Oral Liquid Formulations of Antibiotics

**Figure 9:**
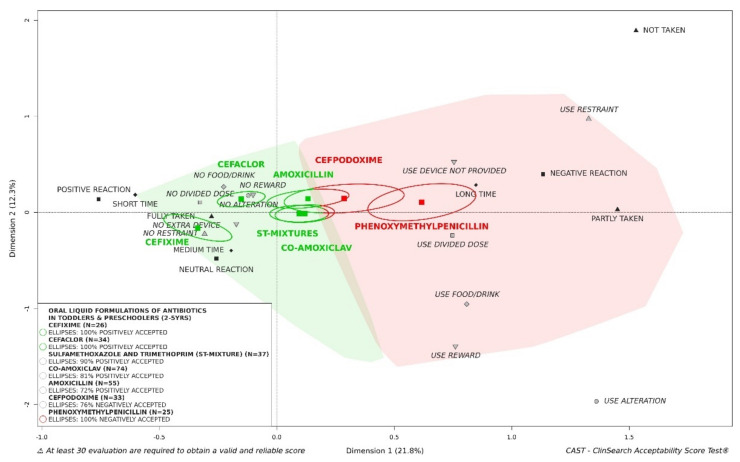
Acceptability of oral liquid formulations of antibiotics in toddlers and preschoolers depending on active pharmaceutical ingredient (API).

As expected, API may also influence acceptability in toddlers and preschoolers (Figure 9). Cefixime tended (*n* < 30) to be the best accepted API. Cefaclor, which was fully located in the green area of the 3D-map, was also positively accepted. Although the barycenters of the evaluations of sulfamethoxazole and trimethoprim (ST-mixture), co-amoxiclav and amoxicillin were located in the green area of the map, those APIs were not classified as accepted due to a part of confidence ellipses in the “negatively accepted” profile. Cefpodoxime was not classified as positively accepted due to a barycenter, and 76% of the confidence ellipsis surrounding it, located the “negatively accepted” profile. Phenoxymethylpenicillin tended (*n* < 30) to be fully located in the red area of the map. Table S10 shows significant differences between the seven API subgroups of evaluations in terms of setting, product features and patient characteristics with the exception of patient sex.

## 4. Discussion

Mining 628 real-life observer reports of the intake of 133 distinct medicines in seven countries, the acceptability reference framework confirmed the influence of many factors on acceptability of oral antibiotics in children. This paper mainly deals with toddlers and preschoolers, an age group of considerably more variability in oral antibiotic acceptability compared to other age groups. The results indicate that changes in acceptability could be due to the context (place of administration), patient characteristics (age group, sex, treatment exposure) and product characteristics (API, pharmaceutical form category, administration device provided and flavor for oral liquids). Findings on those acceptability drivers are discussed hereafter.

An effect of the context was observed in this study. Oral liquid preparations of antibiotics appeared to be better accepted at the hospital than at home in toddlers and preschoolers as well as in grade-schoolers. As data were collected by carers for outpatients and healthcare professionals for inpatients, the satisfactory inter-rater reliability was previously verified to limit bias and ensure consistency of the measures regardless of the observer [33]. The effect of the context observed in this study could be due to healthcare professional skills, or potentially a difference in the study population (e.g., disease severity). However, for newborns and infants contrary findings were obtained. Due to the different APIs used in the hospital vs. home setting (e.g., phenoxymethylpenicillin and cefpodoxime studied mainly at home, and cefaclor studied only in hospital) an effect of the API cannot be excluded. As with any confounding factors, APIs could significantly confound the impact of other parameters, if they were not equally distributed between studied subgroups of evaluations. Assessment on antibiotic acceptability in children at home was performed in France and Norway, while data were collected at hospital in more countries: Morocco, Japan, India, Germany, England, and France. Expanding data collection at home should allow to better grasp the influence of setting across different countries. There were important variations between countries in terms of products and APIs in our dataset—e.g., 96% of evaluations of cefaclor in children under 12 years of age were from Japan, while 82% of evaluations of phenoxymethylpenicillin were from Norway. Investigating the influence of the culture is of real interest, therefore additional data are needed to make relevant comparisons among different countries. However, it may be assumed that cultural drivers could be difficult to decipher as the population is culturally diverse within some countries. Furthermore, inherent differences between countries in terms of API prescription and product characteristics—e.g., flavor composition and strength of co-amoxiclav originator products (Augmentin) change between France and England—can complicate cross-country comparisons.

As might be expected, we clearly established an improvement of acceptability with increasing age of the children. Considered as a whole, antibiotics could not be considered as accepted in newborns and infants, while they appeared to be positively accepted in older children. Furthermore, the acceptability reference framework showed the influence of exposure to treatment. Considered as a whole, the subgroup of evaluations of medicine already taken by the children was classified as “positively accepted” while the subgroup of evaluations of medicine never taken by the children was not similarly classified as accepted. This difference could reflect an element of ‘training’ for the child, but could also be due to carers becoming increasingly used to preparing and administering the medicine, especially at home. Such a finding referred also to neophobia, an important concern in pediatric psychology [34], reflecting a tendency to reject unknown or novel products, particularly food. In this study, oral liquid preparations of antibiotics considered as a whole were not classified as positively accepted in young girls, while they were accepted in boys aged 2 to 5 years. Furthermore, we showed the contrasting effect of lemon flavor: the oral liquid preparations of antibiotics with lemon in the list of excipients were significantly less accepted by girls than by boys. In our investigations in young children, there was always a greater issue with girls when an acceptability difference occurred between both sexes. This echoes with a higher sensitivity among older women to the unpalatable oral liquid products which has been similarly observed in elderly patients [35,36].

These findings indicate the role of administration devices in acceptability, as previously reported [37]. Medicines provided with an oral syringe were significantly better accepted than those provided with a measuring spoon in toddlers and preschoolers. Although it is recognized that using a spoon may lead to inaccurate and variable dosing [38,39], 32% of oral liquid evaluations collected in real-life conditions in toddlers and preschoolers in this study, focused on medicinal products providing with such a device. An effect of the API cannot be excluded, e.g., cefixime which tended to be positively accepted (*n* < 30) was only given with an oral syringe while phenoxymethylpenicillin which tended to be negatively accepted (*n* < 30) was given with a measuring spoon. As expected, acceptability and compliance depend also on the API. Separating the evaluations of oral liquid preparations of antibiotics in acceptability in toddlers and preschoolers, significant differences were seen between the seven APIs most assessed. Using parent-reported outcomes—a five-point facial hedonic scale with anchors—from 1482 patients, Wollner et al. [15] highlighted better acceptability for co-amoxiclav than for cefpodoxime. The acceptability reference framework provided consistent findings as 81% of confidence ellipses were in the “positively accepted” zone for co-amoxiclav and 76% were in the “negatively accepted” zone for cefpodoxime. Using a single-blind taste test—10 cm VAS incorporating a facial hedonic scale—performed by healthy children, Toscani et al. [40] and Angelilli et al. [41] determined palatability differences among flavored antimicrobial agents. The strawberry flavored suspension of cefixime was most commonly rated as the best tasting, compared to banana flavored co-amoxiclav. According to the acceptability reference framework strawberry flavored cefixime tended to be positively acceptability. Co-amoxiclav formulations assessed in this study were not banana flavored. Nevertheless, co-amoxiclav appeared to be significantly less accepted than cefixime. Such a difference was consistent with results from the Gooch et al. study based on parents’ reports [42]. Based on different graded scores reported by phone by parents of 546 children, Dagan et al. [43] classified drugs as follows, from the better to the poorer accepted: cefaclor, amoxicillin, ST-mixture and cefuroxime axetil. The reference framework provided us with concordant findings: cefaclor was better accepted than amoxicillin and ST-mixture. Such findings on cefaclor are consistent with the study of Pichichero et al. [44] which highlighted higher taste acceptability of cefaclor in comparison with amoxicillin-clavulanate potassium and cefuroxime axetil, based on the reaction to the taste of 377 children reported by parents. Although acceptability scores from the reference framework were consistent with other measures from the literature, additional evaluations are needed for cefixime and phenoxymethylpenicillin to get reliable scores with a satisfactory precision using this multivariate approach. Furthermore, there were important differences between evaluations of APIs in our dataset, in terms of setting, patient characteristics and product features.

Allowing comparison of standardized evaluation of acceptability, the reference framework appears to be of real benefit for improving our understanding of acceptability drivers in pediatrics. This study on oral antibiotics in children confirmed that acceptability is likely to be determined by many factors. Based on a significant number of evaluations, relevant acceptability scores were obtained and significant differences were highlighted in this study. However, an effect of confounding factors cannot be excluded for some comparisons. Although some factors were evaluated in this paper, many other characteristics may influence acceptability. For example, excipients such as sweeteners or thickening agents as well as temperature of the preparation may impact palatability beyond API and flavor. Although crucial in pediatrics, palatability is only one facet of acceptability as described in the introduction [16]. Due to the wide range of medicines available on the global market, and the large variety of users worldwide, a comprehensive understanding of acceptability drivers remains challenging. The study dataset was not representative of the world population and the global pharmaceutical market. However, the 3130 standardized evaluations reflect a wide variability of usage that could be observed in real-life conditions with sufficient variations in medicines, users, and settings to ensure a satisfactory reliability of the model as previously demonstrated using resampling methods [26,33]. Studying the combined effect of distinct drivers as well as exploring new factors while reducing the influence of confounding factors by continuously implementing our model with new data should contribute to the evidence base on acceptability drivers of antibiotics in pediatrics.

## 5. Conclusions

Acceptability of antibiotics is crucial to ensure patient adherence in children and consequently, in addition to effective treatment of the infection, is an important contribution to prevent the emergence and dissemination of resistant bacterial strains endangering the efficacy of these pivotal drugs. This international observational study corroborates the multidimensional nature of oral antibiotic acceptability in children. Indeed, the results indicate changes in acceptability due to the context of use (place of administration), patient characteristics (age group, sex, treatment exposure) and product characteristics (API, pharmaceutical form category, administration device provided and flavor for oral liquids). Further explorations are needed to thoroughly evaluate the many acceptability drivers of antibiotics in children. Considering all the many aspects of acceptability to adequately assess this multi-faceted concept, as well as for designing or prescribing medicine in children, is essential to reach adequate acceptability in the target population ensuring patient compliance.

## Figures and Tables

**Table 1:** Characteristics of the patients and products included in the study, stratified by patient age.

Characteristics	Patient Age			Statistical Test
	Newborns and Infants 0–1 Year (*n* = 92)	Toddlers and Preschoolers 2–5 Years (*n* = 372)	Grade-Schoolers 6–11 Years (*n* = 164)	
**Sex**				χ^2 b^: *p* = 0.77
Female	48 (53) ^a^	199 (54)	79 (50)	
Male	43 (47)	171 (46)	78 (50)	
*missing data*	*1*	*2*	*7*	
**Treatment exposure**				χ^2^: *p* < 0.001
Previous exposure	20 (22)	155 (42)	88 (54)	
First exposure	72 (78)	217 (58)	76 (46)	
**Setting**				χ^2^: *p* = 0.007
Community	31 (34)	160 (43)	48 (29)	
Hospital	61 (66)	212 (57)	116 (71)	
**Country**				χ^2^: *p* < 0.001
France	32 (35)	135 (36)	39 (24)	
Morocco	23 (25)	68 (18)	42 (26)	
Japan	18 (20)	71 (19)	36 (22)	
Norway	2 (2)	36 (10)	12 (7)	
India	2 (2)	25 (7)	23 (14)	
Germany	10 (11)	21 (6)	3 (2)	
England	5 (5)	16 (4)	9 (5)	
**5th level (chemical substance) of the Anatomical Therapeutic Chemical (ATC) classification system**				χ^2^: *p* < 0.001
Amoxicillin and clavulanic acid (co-amoxiclav)	20 (22)	75 (20)	25 (15)	
Amoxicillin	17 (18)	57 (15)	20 (12)	
Sulfamethoxazole and trimethoprim (ST-mixture)	19 (21)	41 (11)	25 (15)	
Cefpodoxime	7 (8)	36 (10)	14 (9)	
Cefaclor	2 (2)	34 (9)	17 (10)	
Josamycin	20 (22)	23 (6)	6 (4)	
Cefixime	3 (3)	26 (7)	15 (9)	
Fusidic acid	3 (3)	21 (6)	16 (10)	
Phenoxymethylpenicillin	0 (0)	25 (7)	9 (5)	
Other (<5%)	1 (1)	34 (9)	17 (10)	
**Pharmaceutical form** **categories**				χ^2^: *p* < 0.001
Oral liquids ^c^	92 (100)	357 (96)	137 (84)	
Solid oral dosage form	0 (0)	15 (4)	27 (16)	

^a^*n* (%): number and percentages; ^b^ χ^2^: Pearson’s chi-squared test *p*-value; ^c^ Oral liquids encompass ready to-use oral liquids (i.e., oral solutions or suspensions) as well as reconstituted oral liquids (i.e., powders or effervescent tablets which must be dissolved or dispersed in a liquid prior to administration).

## Data Availability

Data underlying the study cannot be made publicly available due to legal and ethical considerations. European Union (GDPR) and French (Law n°78–17 of 6 January 1978) laws restrict the public sharing of personally identifiable data. Requests for data will be processed according to the French MR-003 Code of conduct by the data controller, ClinSearch, which allows for the use of data for the purpose of reproducing study results. Requests to access the data for this purpose may be sent to the data protection officer of ClinSearch: dataprivacy@clinsearch.net and researchers outside the European Union will need to sign a transfer agreement.

## Supplementary Materials

The following are available online at https://www.mdpi.com/article/10.3390/pharmaceutics13101721/s1, Table S1. Characteristics of the patients and products focusing on oral liquid formulations of antibiotics in toddlers and preschoolers in the study, stratified by administration device provided with the medicine; Table S2. Characteristics of the patients and products focusing on oral liquid formulations of antibiotics in toddlers and preschoolers in the study, stratified by place of administration; Table S3. Characteristics of the patients and products focusing on oral liquid formulations of antibiotics in toddlers and preschoolers in the study, stratified by patient exposure to treatment; Table S4. Characteristics of the patients and products focusing on oral liquid formulations of antibiotics in toddlers and preschoolers in the study, stratified by patient sex; Table S5. Characteristics of the patients and products focusing on oral liquid formulations of co-amoxiclav in toddlers and preschoolers in the study, stratified by patient sex; Table S6. Characteristics of the patients and products focusing on oral liquid formulations of antibiotics with lemon flavor in the list of excipients in toddlers and preschoolers in the study, stratified by patient sex; Table S7. Characteristics of the patients and products focusing on oral liquid formulations of antibiotics with strawberry flavor in the list of excipients in toddlers and preschoolers in the study, stratified by patient sex; Table S8. Characteristics of the patients and products focusing on oral liquid formulations of antibiotics with banana flavor in the list of excipients in toddlers and preschoolers in the study, stratified by patient sex; Table S9. Characteristics of the patients and products focusing on oral liquid formulations of antibiotics with strawberry flavor in the list of excipients in toddlers and preschoolers in the study, stratified by active pharmaceutical ingredient; Table S10. Characteristics of the patients and products focusing on oral liquid formulations of antibiotics in toddlers and preschoolers in the study, stratified by active pharmaceutical ingredient; Figure S1: Acceptability of oral liquid formulations of antibiotics in grade-schoolers depending on administration device; Figure S2: Acceptability of oral liquid formulations of antibiotics in newborns and infants depending on administration device; Figure S3: Acceptability of oral liquid formulations of antibiotics in grade-schoolers depending on place of administration; Figure S4: Acceptability of oral liquid formulations of antibiotics in newborns and infants depending on place of administration; Figure S5: Acceptability of oral liquid formulations of antibiotics in grade-schoolers depending on exposure to treatment; Figure S6: Acceptability of oral liquid formulations of antibiotics in newborns and infants depending on exposure to treatment; Figure S7: Acceptability of oral liquid formulations of antibiotics in newborns and infants depending on sex of patients; Figure S8: Acceptability of oral liquid formulations of antibiotics in grade-schoolers depending on sex of patients; Figure S9: Acceptability of oral liquid formulations of co-amoxiclav in toddlers and preschoolers depending on the sex of patients; Figure S10: Acceptability of oral liquid formulations of antibiotics with strawberry flavor in the list of excipients in toddlers and preschoolers depending on the sex of patients; Figure S11: Acceptability of oral liquid formulations of antibiotics with banana flavor in the list of excipients in toddlers and preschoolers depending on the sex of patients; Figure S12: Acceptability of oral liquid formulations of antibiotics with strawberry flavor in the list of excipients in toddlers and preschoolers depending on API.
