# Supplementary material for: Exploring Acceptability Drivers of Oral Antibiotics in Children: Findings from an International Observational Study
Source: Pharmaceutics. 2021 Oct 18;13(10):1721. doi: 10.3390/pharmaceutics13101721 (PMC8537532; doi:10.3390/pharmaceutics13101721)
Supplement: Supplementary file 1 [file pharmaceutics-13-01721-s001.zip › pharmaceutics-1373668-supplementary.pdf]

pharmaceutics

MDPI

Supplementary Materials: Exploring Acceptability Drivers of Oral Antibiotics in Children: Findings from an International Observational Study

Thibault Vallet, Yahya Bensouda, Jumpei Saito, Liv Mathiesen, Varsha Pokharkar, Viviane Klingmann, Matthew Peak, Omar Elhamdaoui, Akimasa Yamatani, Ivana Ivanovic, Manjusha Sajith, Juliane Münch, Louise Bracken, Jennifer C. Duncan, Smita Salunke, Siri Wang, Fabrice Ruiz

Table S1. Characteristics of the patients and products focusing on oral liquid formulations of antibiotics in toddlers and preschoolers in the study, stratified by administration device.

Characteristics	Oral syringe (n = 129)	Measuring spoon (n = 114)	Statistical Test
**Sex**			$\chi^{2\,a}$: p=.24
Girl	55 (43)	58 (51)	
Boy	73 (57)	55 (49)	
missing data	*1*	*1*	
**Age group**			χ^2: p=.25
Toddlers (2y.)	55 (43)	58 (51)	
Preschoolers (3-5y.)	74 (57)	56 (49)	
**Treatment exposure**			χ^2: p=.08
Previous exposure	51 (40)	59 (52)	
First exposure	78 (60)	55 (48)	
**Setting**			χ^2: p=.002
Community	60 (47)	76 (67)	
Hospital	69 (53)	38 (33)	
**Country**			χ^2: p<.001
France	61 (47)	70 (61)	
Morocco	45 (35)	21 (18)	
Norway	0 (0)	15 (13)	
Germany	18 (14)	3 (3)	
England	5 (4)	5 (4)	
**5th level (chemical substance) of the Anatomical Therapeutic Chemical (ATC) classification system**			χ^2: p<.001
Co-amoxiclav	57 (44)	5 (4)	
Amoxicillin	0 (0)	50 (44)	
Cefpodoxime	16 (12)	16 (14)	
Cefixime	26 (20)	0 (0)	
Josamycin	23 (18)	0 (0)	
Fusidic acid	0 (0)	21 (18)	
Phenoxymethylpenicillin	0 (0)	15 (13)	
Other (<5%)	7 (5)	7 (6)	
**Pharmaceutical form categories**			χ^2: p<.001
Reconstituted oral liquids	129 (100)	86 (75)	
Ready to-use oral liquids	0 (0)	28 (25)	
**Flavor composition**			χ^2: p<.001
Strawberry	68 (53)	0 (0)	
Lemon ; orange ; peach-apricot	18 (14)	26 (23)	

Banana	7 (5)	16 (14)
Banana ; orange	0 (0)	21 (18)
E99 294 55	19 (15)	0 (0)
Lemon ; peach ; strawberry	0 (0)	16 (14)
Dark cherry	0 (0)	13 (11)
Orange ; caramel	0 (0)	12 (11)
Lemon ; orange	9 (7)	0 (0)
Other (<5%)	8 (6)	10 (9)

[a] χ^2: Pearson's Chi-squared Test p-value

Table S2. Characteristics of the patients and products focusing on oral liquid formulations of antibiotics in toddlers and preschoolers in the study, stratified by place of administration.

Characteristics	Hospital (n = 200)	Home (n = 157)	Statistical Test
**Sex**			$\chi^{2\,a}$: *p*=.38
Girl	96 (48)	67 (43)	
Boy	103 (52)	89 (57)	
missing data	*1*	*1*	
**Age group**			χ^2: *p*=.21
Toddlers (2y.)	90 (45)	82 (52)	
Preschoolers (3-5y.)	110 (55)	75 (48)	
**Treatment exposure**			χ^2: *p*=.76
Previous exposure	81 (40)	67 (43)	
First exposure	119 (60)	90 (57)	
**Country**			χ^2: *p*<.001
France	10 (5)	123 (78)	
Morocco	68 (34)	0 (0)	
Japan	70 (35)	0 (0)	
Norway	0 (0)	34 (22)	
India	15 (8)	0 (0)	
Germany	21 (10)	0 (0)	
England	16 (8)	0 (0)	
**5th level (chemical substance) of the Anatomical Therapeutic Chemical (ATC) classification system**			χ^2: *p*<.001
Co-amoxiclav	42 (21)	32 (20)	
Amoxicillin	12 (6)	43 (27)	
Sulfamethoxazole and trimethoprim	34 (17)	3 (2)	
Cefaclor	34 (17)	0 (0)	
Cefpodoxime	1 (0)	32 (20)	
Cefixime	21 (10)	5 (3)	
Phenoxymethylpenicillin	3 (2)	22 (14)	
Josamycin	17 (8)	6 (4)	
Fusidic acid	21 (10)	0 (0)	
Other (<5%)	15 (8)	14 (9)	
**Pharmaceutical form categories**			χ^2: *p*<.001
Reconstituted oral liquids	153 (76)	153 (97)	
Ready to-use oral liquids	47 (24)	4 (3)	
**Provided administration device**			F^b: *p*<.001
Oral syringe	69 (36)	60 (44)	
Measuring spoon	38 (20)	76 (55)	
Device not provided	71 (37)	0 (0)	
Measuring cup	15 (8)	0 (0)	
Other (<5%)	0 (0)	1 (1)	
missing data	*7*	*20*	
**Flavor composition**			χ^2: *p*<.001
Strawberry	49 (25)	23 (15)	
No flavour	45 (23)	0 (0)	
Lemon ; orange ; peach-apricot	30 (15)	14 (9)	
Orange	34 (18)	2 (1)	

Banana	0 (0)	23 (15)
Banana ; orange	21 (11)	0 (0)
Lemon ; peach ; strawberry	2 (1)	17 (11)
E99 294 55	1 (1)	18 (11)
Dark cherry	0 (0)	13 (8)
Orange ; caramel	0 (0)	12 (8)
Lemon ; orange	0 (0)	9 (6)
Lemon ; peach ; strawberry	3 (7)	0 (0)
Other (<5%)	12 (6)	18 (11)
missing data	6	0

[a] χ^2: Pearson's Chi-squared Test p-value ; [b] F: Fisher's exact test p-value

pharmaceutics MDPI

Table S3. Characteristics of the patients and products focusing on oral liquid formulations of antibiotics in toddlers and preschoolers in the study, stratified by patient exposure to treatment.

Characteristics	Previous exposure (n = 148)	First exposure (n = 209)	Statistical Test
**Sex**			$\chi^{2\,a}$: *p*=1
Girl	67 (46)	96 (46)	
Boy	80 (54)	112 (54)	
missing data	*1*	*1*	
**Age group**			χ^2: *p*=.21
Toddlers (2y.)	65 (44)	107 (51)	
Preschoolers (3-5y.)	83 (56)	102 (49)	
**Setting**			χ^2: *p*=.76
Community	67 (45)	90 (43)	
Hospital	81 (55)	119 (57)	
**Country**			χ^2: *p*<.001
France	71 (48)	62 (30)	
Morocco	29 (20)	39 (19)	
Japan	27 (18)	43 (21)	
Norway	6 (4)	28 (13)	
India	0 (0)	15 (7)	
Germany	0 (0)	21 (10)	
England	15 (10)	1 (0)	
**5th level (chemical substance) of the Anatomical Therapeutic Chemical (ATC) classification system**			χ^2: *p*<.001
Co-amoxiclav	40 (19)	34 (23)	
Amoxicillin	27 (13)	28 (19)	
Sulfamethoxazole and trimethoprim	11 (5)	26 (18)	
Cefaclor	30 (14)	4 (3)	
Cefpodoxime	18 (9)	15 (10)	
Cefixime	16 (8)	10 (7)	
Phenoxymethylpenicillin	19 (9)	6 (4)	
Josamycin	16 (8)	7 (5)	
Fusidic acid	11 (5)	10 (7)	
Other (<5%)	21 (10)	8 (5)	
**Pharmaceutical form categories**			χ^2: *p*=.91
Reconstituted oral liquids	180 (86)	126 (85)	
Ready to-use oral liquids	29 (14)	22 (15)	
**Provided administration device**			F^b: *p*<.001
Oral syringe	51 (37)	78 (40)	
Measuring spoon	59 (43)	55 (28)	
Device not provided	27 (20)	44 (23)	
Measuring cup	0 (0)	15 (8)	
Other (<5%)	0 (0)	1 (1)	
missing data	*11*	*16*	
**Flavor composition**			χ^2: *p*=.001
Strawberry	28 (20)	44 (21)	
No flavour	23 (16)	22 (11)	
Lemon ; orange ; peach-apricot	18 (13)	26 (12)	
Orange	5 (4)	31 (15)	

Banana	12 (8)	11 (5)
Banana ; orange	10 (7)	11 (5)
Lemon ; peach ; strawberry	10 (7)	9 (4)
E99 294 55	13 (9)	6 (3)
Orange ; caramel	1 (1)	11 (5)
Other (<5%)	22 (15)	38 (18)
missing data	*6*	*0*

[a] χ^2: Pearson's Chi-squared Test p-value ; [b] F: Fisher's exact test p-value

Table S4. Characteristics of the patients and products focusing on oral liquid formulations of antibiotics in toddlers and preschoolers in the study, stratified by patient sex.

Characteristics	Boys (n = 192)	Girls (n = 163)	Statistical Test
**Age group**			χ^{2a}: p=.83
Toddlers (2y.)	91 (47)	80 (49)	
Preschoolers (3-5y.)	101 (53)	83 (51)	
**Treatment exposure**			χ^2: p=1
Previous exposure	80 (42)	67 (41)	
First exposure	112 (58)	96 (59)	
**Setting**			χ^2: p=.38
Community	89 (46)	67 (41)	
Hospital	103 (54)	96 (59)	
**Country**			χ^2: p=.75
France	74 (39)	58 (36)	
Morocco	33 (17)	34 (21)	
Japan	42 (22)	28 (17)	
Norway	18 (9)	16 (10)	
India	6 (3)	9 (6)	
Germany	10 (5)	11 (7)	
England	9 (5)	7 (4)	
**5th level (chemical substance) of the Anatomical Therapeutic Chemical (ATC) classification system**			χ^2: p=.024
Co-amoxiclav	44 (23)	30 (18)	
Amoxicillin	29 (15)	25 (15)	
Sulfamethoxazole and trimethoprim	18 (9)	19 (12)	
Cefaclor	26 (14)	8 (5)	
Cefpodoxime	17 (9)	16 (10)	
Cefixime	16 (8)	10 (6)	
Phenoxymethylpenicillin	15 (8)	10 (6)	
Josamycin	11 (6)	11 (7)	
Fusidic acid	7 (4)	14 (9)	
Other (<5%)	9 (5)	20 (12)	
**Pharmaceutical form categories**			χ^2: p=.014
Reconstituted oral liquids	173 (90)	131 (80)	
Ready to-use oral liquids	19 (10)	32 (20)	
**Provided administration device**			F^b: p=.23
Oral syringe	73 (41)	55 (36)	
Measuring spoon	55 (31)	58 (38)	
Device not provided	43 (24)	28 (19)	
Measuring cup	6 (3)	9 (6)	
Other (<5%)	0 (0)	1 (1)	
missing data	*15*	*12*	
**Flavor composition**			χ^2: p=.014
Strawberry	42 (22)	29 (18)	
No flavour	21 (11)	24 (15)	
Lemon ; orange ; peach-apricot	19 (10)	24 (15)	
Orange	27 (14)	9 (6)	
Banana	11 (6)	12 (8)	

Banana ; orange	7 (4)	14 (9)
Lemon ; peach ; strawberry	15 (8)	4 (2)
E99 294 55	11 (6)	8 (5)
Other (<5%)	36 (19)	36 (22)
missing data	*3*	*3*

[a] χ^2: Pearson's Chi-squared Test p-value ; [b] F: Fisher's exact test p-value

Table S5. Characteristics of the patients and products focusing on oral liquid formulations of co-amoxiclav in toddlers and preschoolers in the study, stratified by patient sex.

Characteristics	Boys (n = 44)	Girls (n = 30)	Statistical Test
**Age group**			χ^{2a}: *p*=.48
Toddlers (2y.)	20 (45)	17 (57)	
Preschoolers (3-5y.)	24 (55)	13 (43)	
**Treatment exposure**			χ^2: *p*=.89
Previous exposure	21 (48)	13 (43)	
First exposure	23 (52)	17 (57)	
**Setting**			χ^2: *p*=.48
Community	21 (48)	11 (37)	
Hospital	23 (52)	19 (63)	
**Country**			F^b: *p*=.16
France	21 (48)	12 (40)	
Morocco	8 (18)	1 (3)	
India	1 (2)	3 (10)	
Germany	9 (20)	9 (30)	
England	5 (11)	5 (17)	
**Pharmaceutical form categories**			χ^2: *p*=.18
Reconstituted oral liquids	41 (93)	24 (80)	
Ready to-use oral liquids	3 (7)	6 (20)	
**Provided administration device**			F^b: *p*=.46
Oral syringe	35 (88)	22 (81)	
Measuring spoon	3 (8)	2 (7)	
Measuring cup	1 (2)	3 (11)	
Other (<5%)	1 (2)	0 (0)	
missing data	*4*	*3*	
**Flavor composition**			F: *p*=.23
Lemon ; orange ; peach-apricot	12 (29)	11 (41)	
Strawberry	15 (36)	5 (19)	
E99 294 55	11 (26)	8 (30)	
No flavour	1 (2)	2 (7)	
Lemon ; peach ; strawberry	3 (7)	0 (0)	
Other (<5%)	0 (0)	1 (4)	
missing data	2	3	

[a] χ^2: Pearson's Chi-squared Test p-value ; [b] F: Fisher's exact test p-value

Table S6. Characteristics of the patients and products focusing on oral liquid formulations of antibiotics with lemon flavor in the list of excipients in toddlers and preschoolers in the study, stratified by patient sex.

Characteristics	Boys (n = 39)	Girls (n = 32)	Statistical Test
**Age group**			χ^2: *p*=.86
Toddlers (2y.)	20 (51)	18 (56)	
Preschoolers (3-5y.)	19 (49)	14 (44)	
**Treatment exposure**			χ^2: *p*=.62
Previous exposure	18 (46)	12 (38)	
First exposure	21 (54)	20 (62)	
**Setting**			χ^2: *p*=.61
Community	23 (59)	16 (50)	
Hospital	16 (41)	16 (50)	
**Country**			χ^2: *p*=.7
France	24 (62)	19 (59)	
Morocco	2 (5)	0 (0)	
Germany	10 (26)	11 (34)	
England	3 (8)	2 (6)	
**5th level (chemical substance) of the Anatomical Therapeutic Chemical (ATC) classification system**			χ^2: *p*=.93
Amoxicillin	19 (49)	17 (53)	
Co-amoxiclav	15 (38)	11 (34)	
Cefpodoxime	5 (13)	4 (12)	
**Pharmaceutical form categories**			
Reconstituted oral liquids	39 (100)	32 (100)	
**Provided administration device**			F[b]: *p*=1
Measuring spoon	22 (59)	19 (59)	
Oral syringe	14 (38)	13 (41)	
Other (<5%)	1 (3)	0 (0)	
missing data	*2*	*0*	
**Flavor composition**			χ^2: *p*=.04
Lemon ; orange ; peach-apricot	19 (49)	24 (75)	
Lemon ; peach ; strawberry	15 (38)	4 (12)	
Lemon ; orange	5 (13)	4 (12)	

[a] χ^2: Pearson's Chi-squared Test p-value ; [b] F: Fisher's exact test p-value

Table S7. Characteristics of the patients and products focusing on oral liquid formulations of antibiotics with strawberry flavor in the list of excipients in toddlers and preschoolers in the study, stratified by patient sex.

Characteristics	Boys (n = 57)	Girls (n = 35)	Statistical Test
**Age group**			χ^{2a}: p=.93
Toddlers (2y.)	23 (40)	13 (37)	
Preschoolers (3-5y.)	34 (60)	22 (63)	
**Treatment exposure**			χ^2: p=.08
Previous exposure	28 (49)	10 (29)	
First exposure	29 (51)	25 (71)	
**Setting**			χ^2: p=.05
Community	31 (54)	11 (31)	
Hospital	26 (46)	24 (69)	
**Country**			F[b]: p=.012
France	31 (54)	11 (31)	
Morocco	26 (46)	20 (57)	
Japan	0 (0)	3 (9)	
India	0 (0)	1 (3)	
**5th level (chemical substance) of the Anatomical Therapeutic Chemical (ATC) classification system**			F: p=.05
Cefixime	16 (28)	10 (29)	
Co-amoxiclav	18 (32)	6 (17)	
Josamycin	11 (19)	11 (31)	
Amoxicillin	12 (21)	4 (11)	
Cefalexin	0 (0)	3 (9)	
Other (<5%)	0 (0)	1 (3)	
**Pharmaceutical form categories**			F: p=.38
Reconstituted oral liquids	57 (100)	34 (97)	
Ready to-use oral liquids	0 (0)	1 (3)	
**Provided administration device**			F: p=.07
Oral syringe	42 (76)	26 (74)	
Measuring spoon	12 (22)	4 (11)	
Device not provided	1 (2)	3 (9)	
Other (<5%)	0 (0)	2 (6)	
missing data	*2*	*0*	
**Flavor composition**			F: p=.05
Strawberry	42 (74)	29 (83)	
Lemon ; peach ; strawberry	15 (26)	4 (11)	
Other flavour label (<5%)	0 (0)	2 (6)	

[a] χ^2: Pearson's Chi-squared Test p-value ; [b] F: Fisher's exact test p-value

pharmaceutics

MDPI

Table S8. Characteristics of the patients and products focusing on oral liquid formulations of antibiotics with banana flavor in the list of excipients in toddlers and preschoolers in the study, stratified by patient sex.

Characteristics	Boys (n = 19)	Girls (n = 29)	Statistical Test
**Age group**			χ^{2a}: *p*=1
Toddlers (2y.)	10 (53)	15 (52)	
Preschoolers (3-5y.)	9 (47)	14 (48)	
**Treatment exposure**			χ^2: *p*=.29
Previous exposure	11 (58)	11 (38)	
First exposure	8 (42)	18 (62)	
**Setting**			χ^2: *p*=.63
Community	12 (63)	15 (52)	
Hospital	7 (37)	14 (48)	
**Country**			F^b: *p*=.78
France	11 (58)	14 (48)	
Morocco	7 (37)	14 (48)	
Norway	1 (5)	1 (3)	
**5th level (chemical substance) of the Anatomical Therapeutic Chemical (ATC) classification system**			F: *p*=.2
Cefpodoxime	11 (58)	12 (41)	
Fusidic acid	7 (37)	14 (48)	
Azithromycin	0 (0)	3 (10)	
Sulfamethoxazole and trimethoprim	1 (5)	0 (0)	
**Pharmaceutical form categories**			χ^2: *p*=.9
Reconstituted oral liquids	11 (58)	15 (52)	
Ready to-use oral liquids	8 (42)	14 (48)	
**Provided administration device**			χ^2: *p*=.99
Measuring spoon	15 (83)	22 (79)	
Oral syringe	3 (17)	6 (21)	
missing data	*1*	*1*	
**Flavor composition**			F: *p*=.21
Banana	11 (58)	12 (41)	
Banana ; orange	7 (37)	14 (48)	
Banana ; cherry ; vanilla	0 (0)	3 (10)	
Banana ; vanilla	1 (5)	0 (0)	

[a] χ^2: Pearson's Chi-squared Test p-value ; [b] F: Fisher's exact test p-value

Table S9. Characteristics of the patients and products focusing on oral liquid formulations of antibiotics with strawberry flavor in the list of excipients in toddlers and preschoolers in the study, stratified by active pharmaceutical ingredient.

Characteristics	Cefixime (n = 26)	Josamycin (n = 23)	Co-amoxiclav (n = 24)	Statistical Test
**Sex**				$\chi^{2\,a}$: p=.22
Girl	10 (38)	11 (50)	6 (25)	
Boy	16 (62)	11 (50)	18 (75)	
missing data	*0*	*1*	*0*	
**Age group**				χ^2: p=.22
Toddlers (2y.)	6 (23)	9 (39)	11 (46)	
Preschoolers (3-5y.)	20 (77)	14 (61)	13 (54)	
**Treatment exposure**				χ^2: p=.39
Previous exposure	10 (38)	7 (30)	12 (50)	
First exposure	16 (62)	16 (70)	12 (50)	
**Setting**				χ^2: p=.009
Community	5 (19)	6 (26)	14 (58)	
Hospital	21 (81)	17 (74)	10 (42)	
**Country**				F^b: p=.008
France	5 (19)	6 (26)	14 (58)	
Morocco	21 (81)	17 (74)	9 (38)	
India	0 (0)	0 (0)	1 (4)	
**Pharmaceutical form categories**				F: p=.64
Reconstituted oral liquids	26 (100)	23 (100)	23 (96)	
Ready to-use oral liquids	0 (0)	0 (0)	1 (4)	
**Provided administration device**				F: p=.09
Oral syringe	26 (100)	23 (100)	20 (91)	
Measuring cup	0 (0)	0 (0)	1 (5)	
Device not provided	0 (0)	0 (0)	1 (5)	
missing data	*0*	*0*	*2*	
**Flavor composition**				F: p=.03
Strawberry	26 (100)	23 (100)	20 (83)	
Lemon ; peach ; strawberry	0 (0)	0 (0)	3 (12)	
Caramel ; strawberry	0 (0)	0 (0)	1 (4)	

[a] χ^2: Pearson's Chi-squared Test p-value ; [b] F: Fisher's exact test p-value

Table S10. Characteristics of the patients and products focusing on oral liquid formulations of antibiotics in toddlers and preschoolers in the study, stratified by active pharmaceutical ingredient.

Characteristics	Cefixime (n = 26)	Cefaclor (n = 34)	ST-mixture[a] (n = 37)	Co-amoxiclav (n = 74)	Amoxicillin (n = 55)	Cefpodoxime (n = 33)	Phenoxymethylpenicillin (n = 25)	Statistical Test
**Sex**								$\chi^{2\,b}$: *p*=.29
Girl	10 (38)	8 (24)	19 (51)	30 (41)	25 (46)	16 (48)	10 (40)	
Boy	16 (62)	26 (76)	18 (49)	44 (59)	29 (54)	17 (52)	15 (60)	
missing data	*0*	*0*	*0*	*0*	*1*	*0*	*0*	
**Age group**								χ^2: *p*=.005
Toddlers (2y.)	6 (23)	10 (29)	21 (57)	37 (50)	30 (55)	19 (58)	17 (68)	
Preschoolers (3-5y.)	20 (77)	24 (71)	16 (43)	37 (50)	25 (45)	14 (42)	8 (32)	
**Treatment exposure**								χ^2: *p*<.001
Previous exposure	10 (38)	4 (12)	26 (70)	34 (46)	28 (51)	15 (45)	6 (24)	
First exposure	16 (62)	30 (88)	11 (30)	40 (54)	27 (49)	18 (55)	19 (76)	
**Setting**								χ^2: *p*<.001
Community	5 (19)	0 (0)	3 (8)	32 (43)	43 (78)	32 (97)	22 (88)	
Hospital	21 (81)	34 (100)	34 (92)	42 (57)	12 (22)	1 (3)	3 (12)	
**Country**								F[c]: *p*<.001
France	5 (19)	0 (0)	4 (11)	33 (45)	47 (85)	32 (97)	3 (12)	
Morocco	21 (81)	0 (0)	0 (0)	9 (12)	0 (0)	0 (0)	0 (0)	
Japan	0 (0)	34 (100)	31 (84)	0 (0)	2 (4)	0 (0)	0 (0)	
Norway	0 (0)	0 (0)	1 (3)	0 (0)	0 (0)	0 (0)	22 (88)	
India	0 (0)	0 (0)	1 (3)	4 (5)	2 (4)	1 (3)	0 (0)	
Germany	0 (0)	0 (0)	0 (0)	18 (24)	3 (5)	0 (0)	0 (0)	
England	0 (0)	0 (0)	0 (0)	10 (14)	1 (2)	0 (0)	0 (0)	
**Pharmaceutical form categories**								F: *p*=.032
Reconstituted oral liquids	26 (100)	34 (100)	31 (84)	65 (88)	52 (95)	32 (97)	22 (88)	
Ready to-use oral liquids	0 (0)	0 (0)	6 (16)	9 (12)	3 (5)	1 (3)	3 (12)	
**Provided administration device**								F: *p*<.001
Oral syringe	26 (100)	0 (0)	0 (0)	57 (85)	0 (0)	16 (48)	0 (0)	
Measuring spoon	0 (0)	0 (0)	4 (11)	5 (7)	50 (93)	16 (48)	15 (100)	
Device not provided	0 (0)	34 (100)	31 (86)	1 (1)	2 (4)	0 (0)	0 (0)	
Measuring cup	0 (0)	0 (0)	1 (3)	4 (6)	2 (4)	1 (3)	0 (0)	
missing data	*0*	*0*	*1*	*7*	*1*	*0*	*10*	
**Flavor composition**								F: *p*<.001
Strawberry	26 (100)	0 (0)	0 (0)	20 (29)	0 (0)	0 (0)	0 (0)	
Lemon ; orange ; peach-apricot	0 (0)	0 (0)	0 (0)	23 (33)	21 (39)	0 (0)	0 (0)	
No flavour	0 (0)	0 (0)	32 (86)	3 (4)	2 (4)	1 (3)	0 (0)	
Orange	0 (0)	34 (100)	0 (0)	0 (0)	0 (0)	0 (0)	2 (8)	
Banana	0 (0)	0 (0)	0 (0)	0 (0)	0 (0)	23 (70)	0 (0)	
Lemon ; peach ; strawberry	0 (0)	0 (0)	0 (0)	3 (4)	16 (30)	0 (0)	0 (0)	

E99 294 55	0 (0)	0 (0)	0 (0)	19 (28)	0 (0)	0 (0)	0 (0)
Dark cherry	0 (0)	0 (0)	0 (0)	0 (0)	13 (24)	0 (0)	0 (0)
Orange ; caramel	0 (0)	0 (0)	0 (0)	0 (0)	0 (0)	0 (0)	12 (48)
Lemon ; orange	0 (0)	0 (0)	0 (0)	0 (0)	0 (0)	9 (27)	0 (0)
Apricot ; grapefruit	0 (0)	0 (0)	0 (0)	0 (0)	0 (0)	0 (0)	8 (32)
Eggs cream ; tutti frutti	0 (0)	0 (0)	4 (11)	0 (0)	0 (0)	0 (0)	0 (0)
Apricot	0 (0)	0 (0)	0 (0)	0 (0)	0 (0)	0 (0)	3 (12)
Other (<5%)	0 (0)	0 (0)	1 (3)	1 (1)	2 (4)	0 (0)	0 (0)
missing data	*0*	*0*	*0*	*5*	*1*	*0*	*0*

[a] ST-mixutre: Sulfamethoxazole and trimethoprim ; [b] χ^2: Pearson's Chi-squared Test p-value ; [c] F: Fisher's exact test p-value

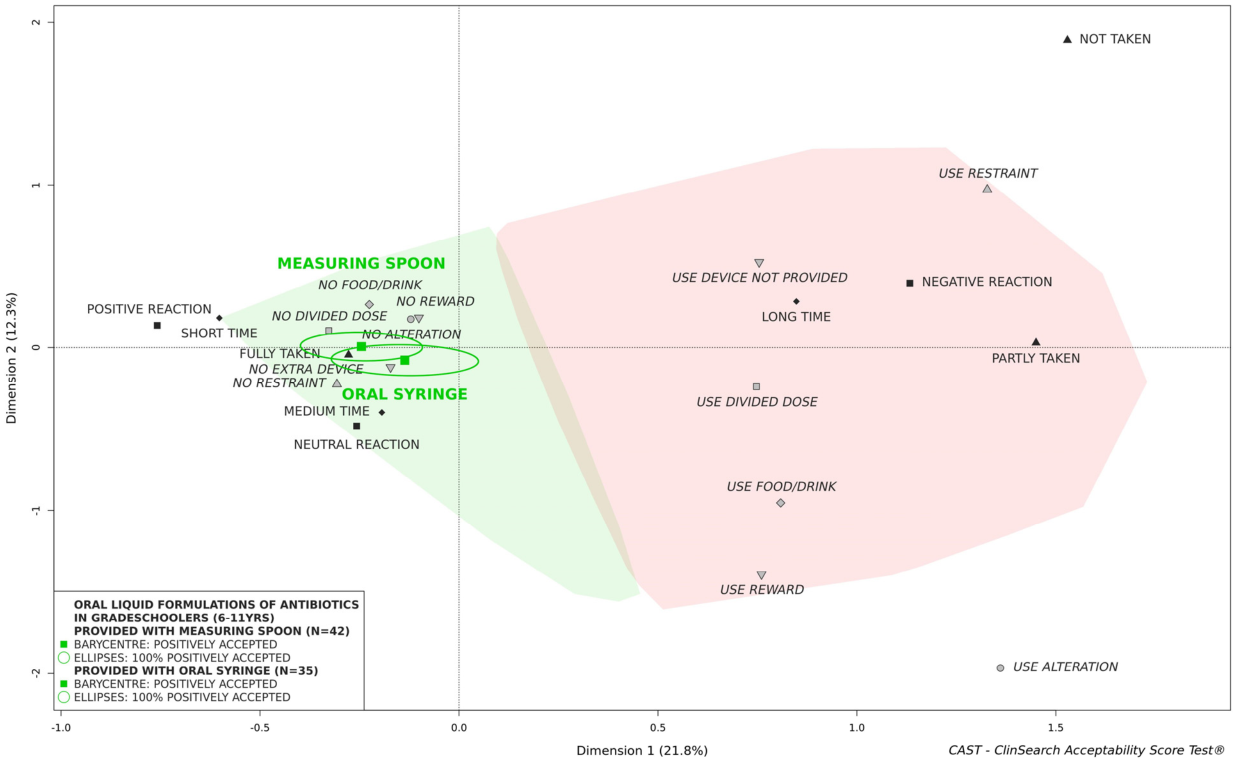

Figure S1. Acceptability of oral liquid formulations of antibiotics in grade-schoolers depending on administration device.

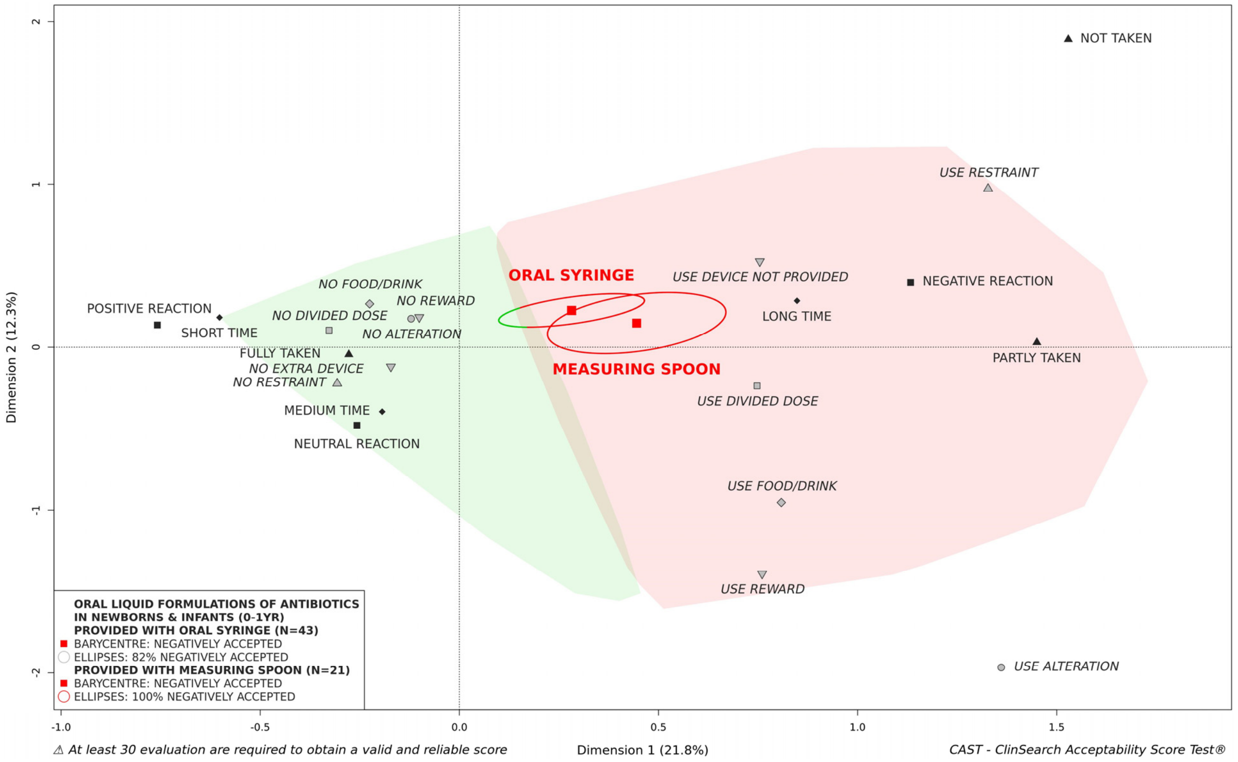

Figure S2. Acceptability of oral liquid formulations of antibiotics in newborns and infants depending on administration device.

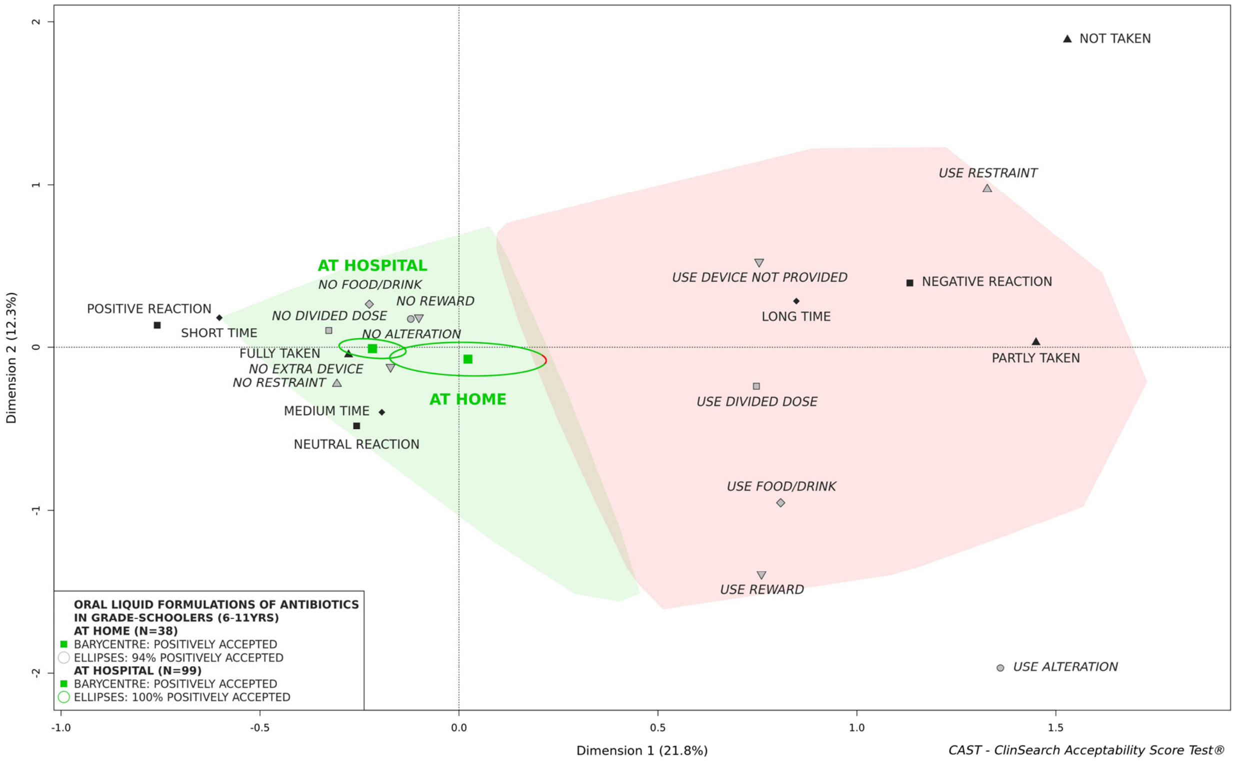

Figure S3. Acceptability of oral liquid formulations of antibiotics in grade-schoolers depending on place of administration.

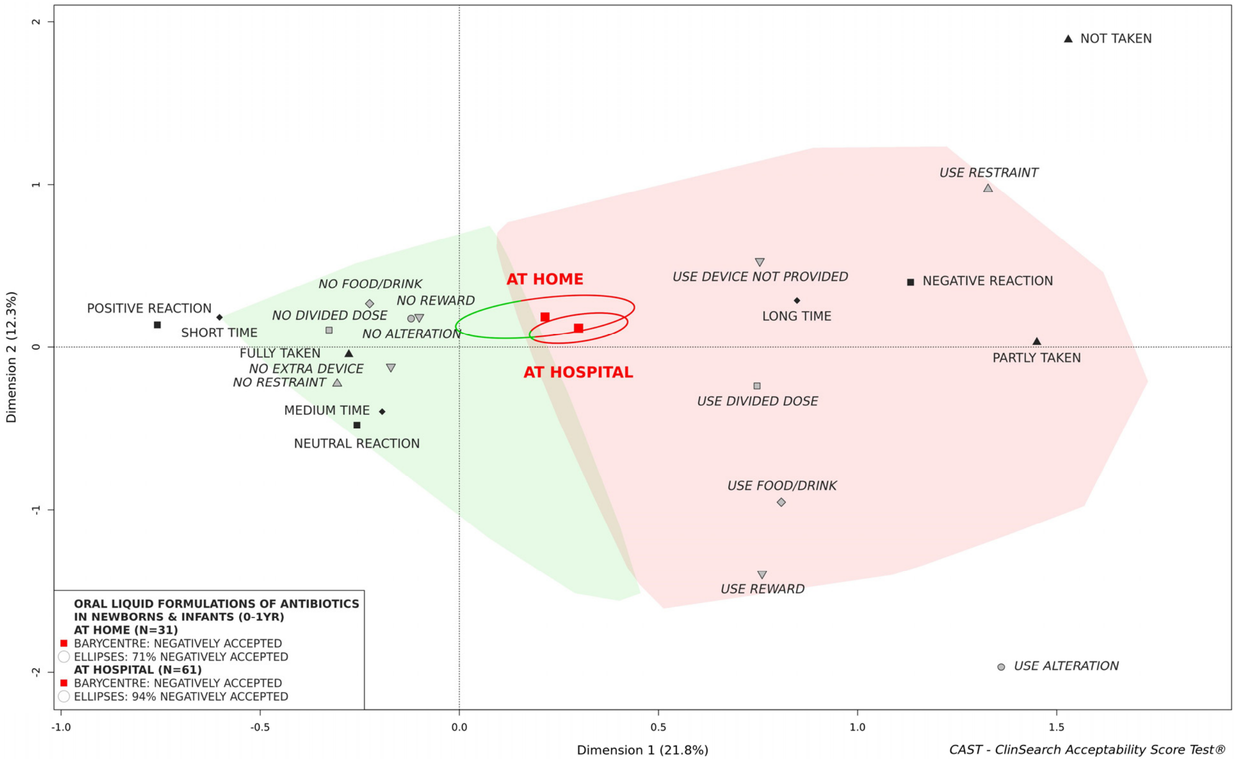

Figure S4. Acceptability of oral liquid formulations of antibiotics in newborns and infants depending on place of administration.

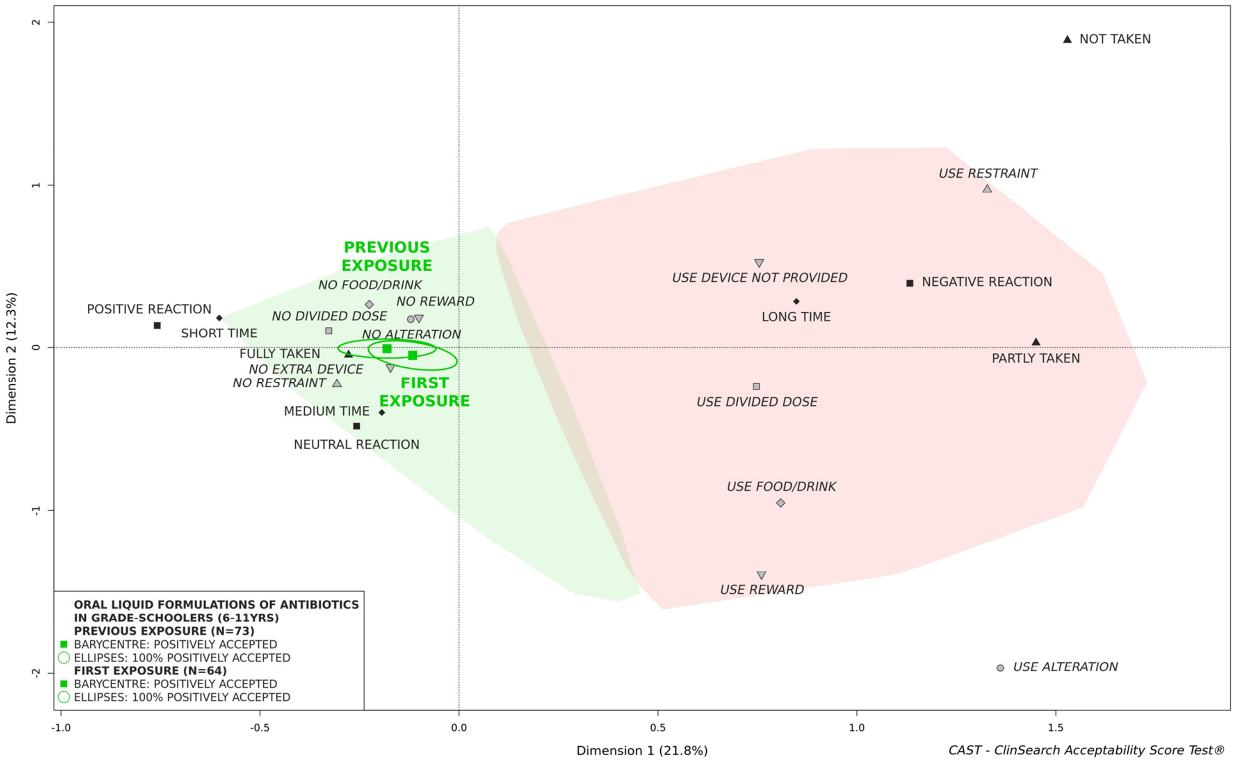

Figure S5. Acceptability of oral liquid formulations of antibiotics in grade-schoolers depending on exposure to treatment.

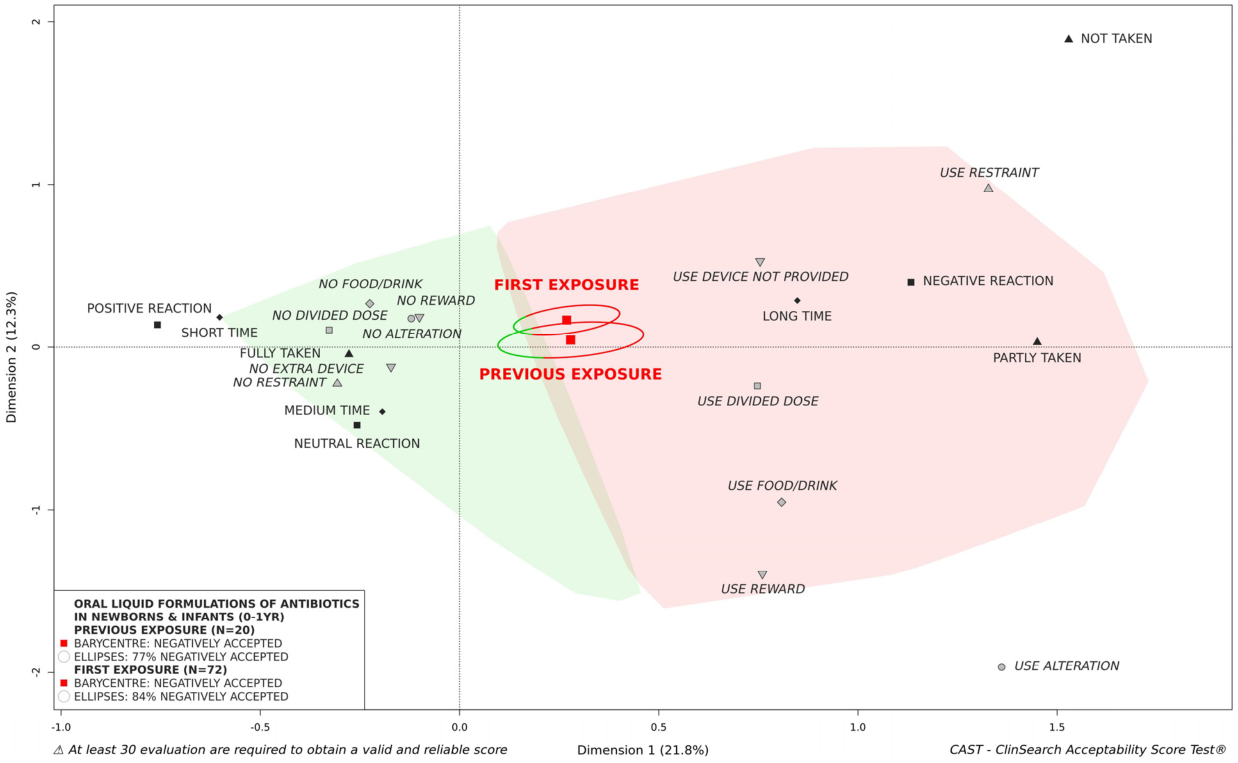

Figure S6. Acceptability of oral liquid formulations of antibiotics in newborns and infants depending on exposure to treatment.

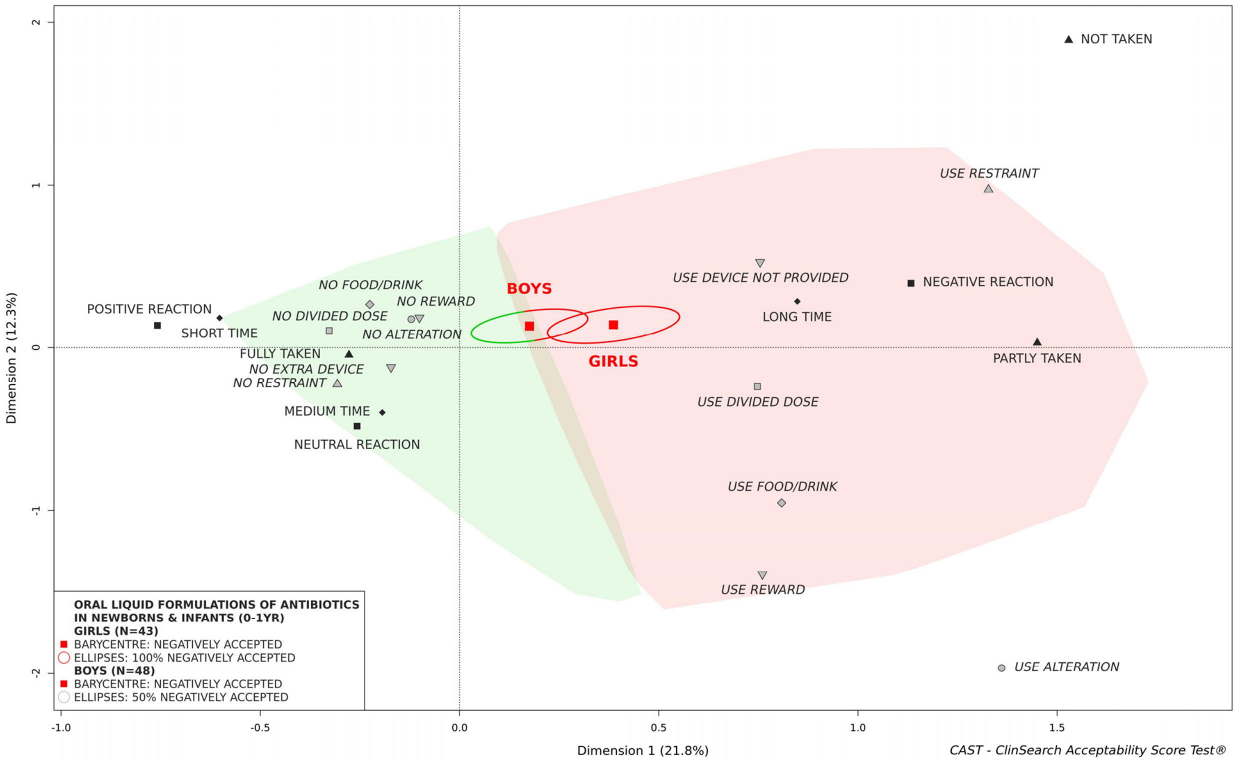

Figure S7. Acceptability of oral liquid formulations of antibiotics in newborns and infants depending on sex of patients.

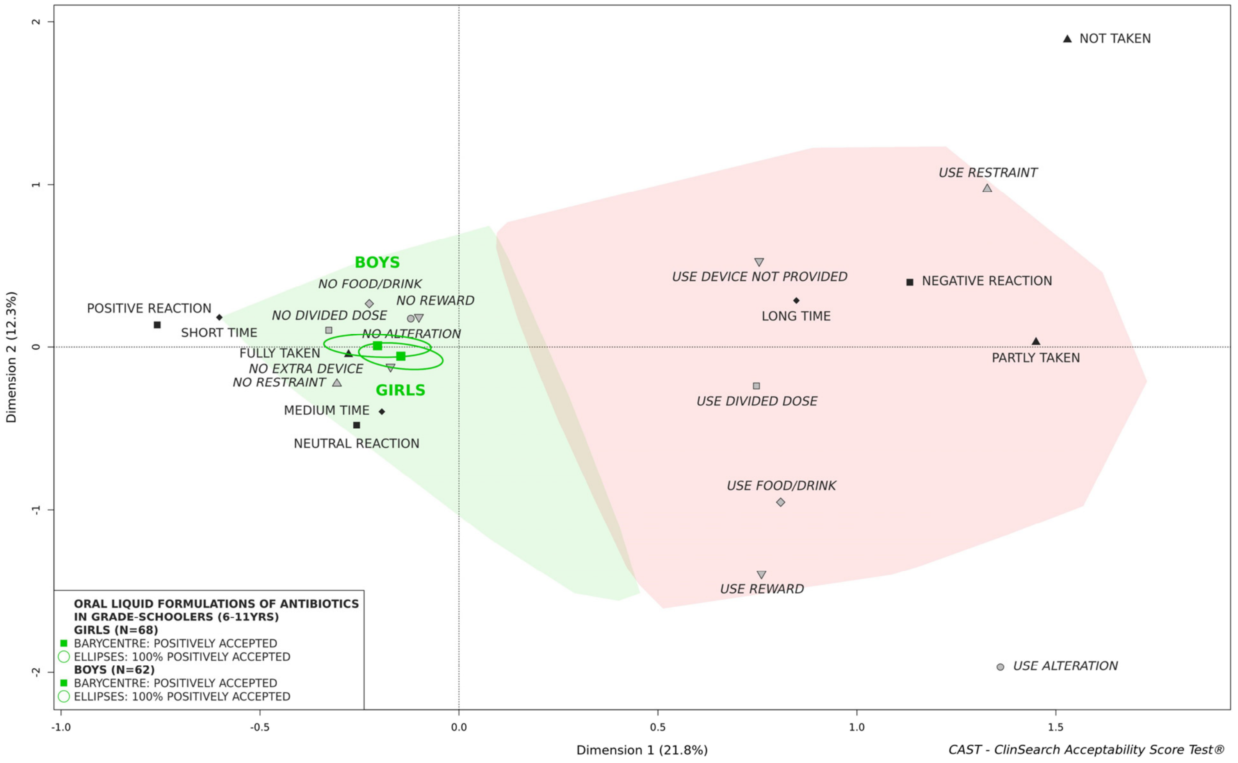

Figure S8. Acceptability of oral liquid formulations of antibiotics in grade-schoolers depending on sex of patients.

pharmaceutics

MDPI

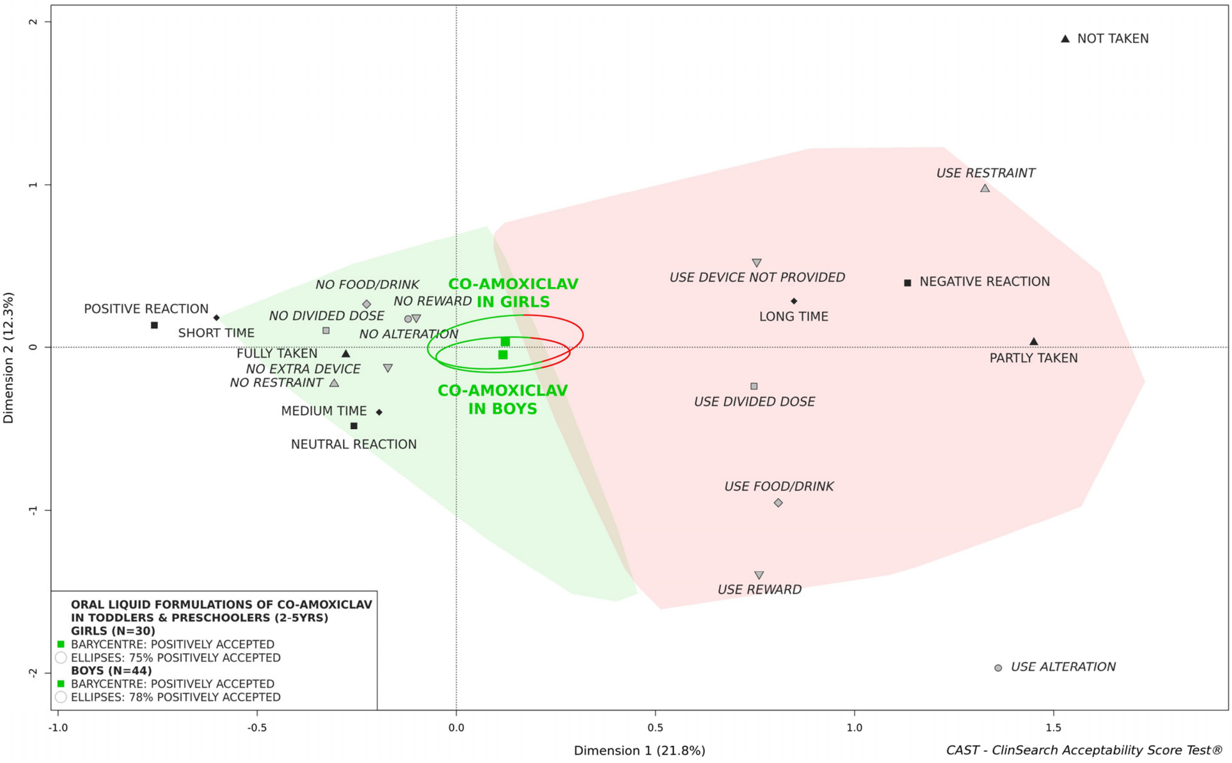

Figure S9. Acceptability of oral liquid formulations of co-amoxiclav in toddlers and preschoolers depending on the sex of patients.

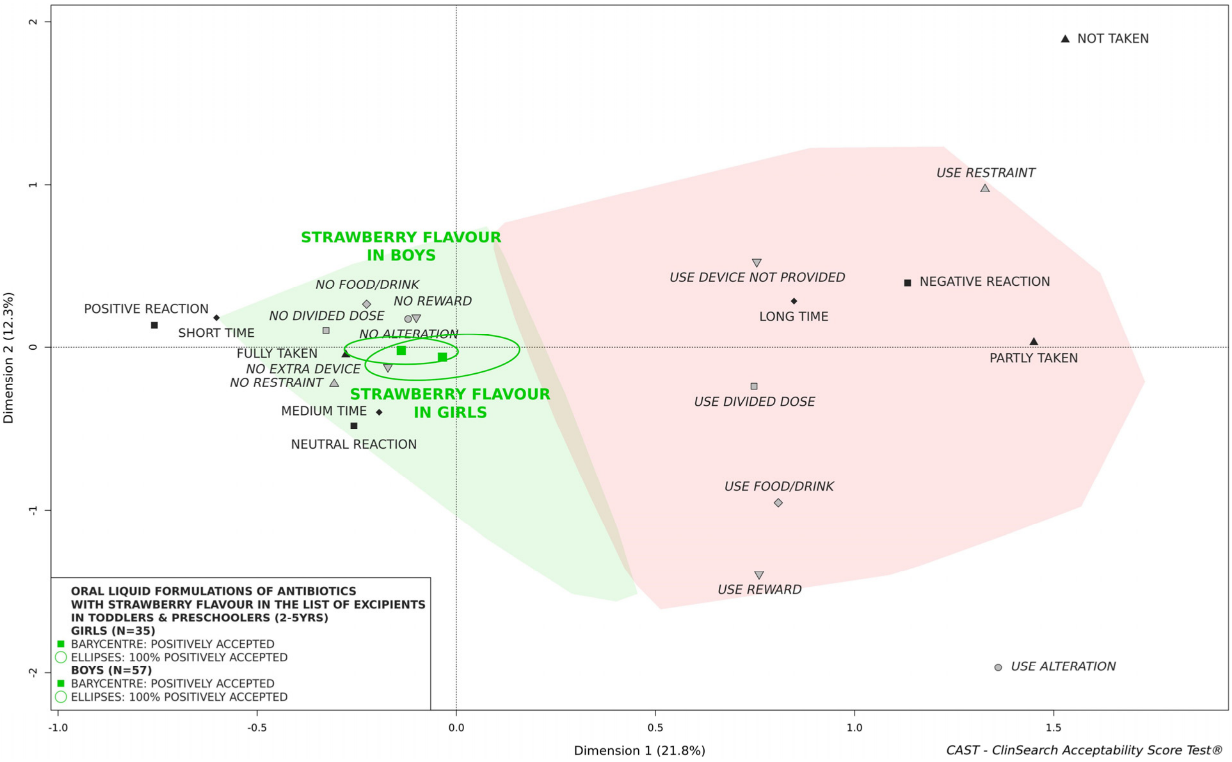

Figure S10. Acceptability of oral liquid formulations of antibiotics with strawberry flavor in the list of excipients in toddlers and preschoolers depending on the sex of patients.

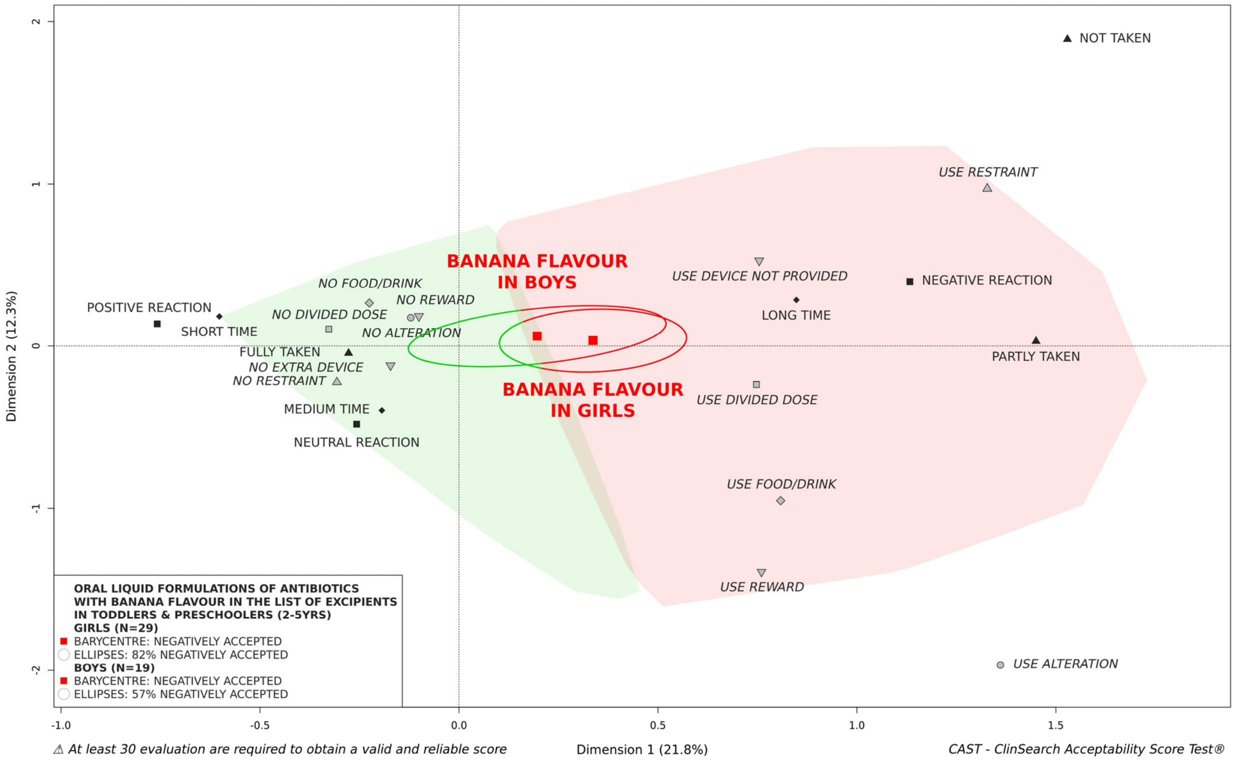

Figure S11. Acceptability of oral liquid formulations of antibiotics with banana flavor in the list of excipients in toddlers and preschoolers depending on the sex of patients.

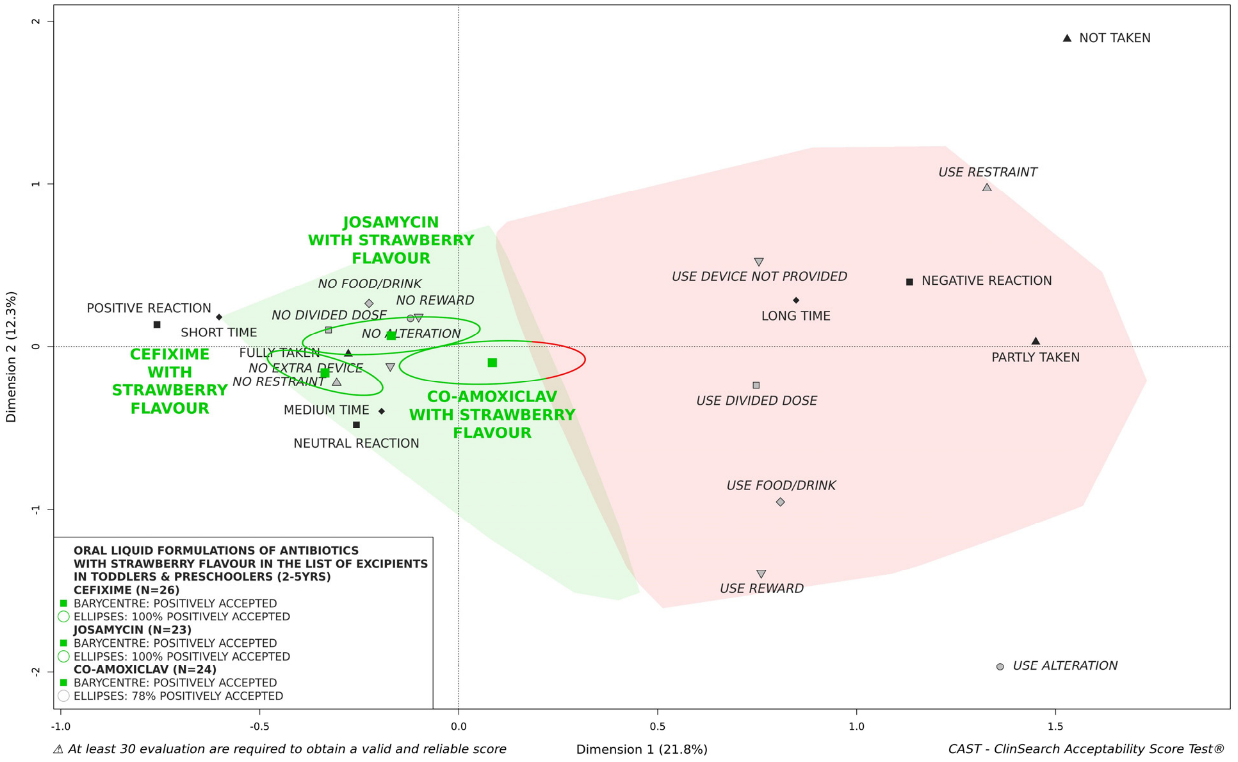

Figure S12. Acceptability of oral liquid formulations of antibiotics with strawberry flavor in the list of excipients in toddlers and preschoolers depending on active pharmaceutical ingredient.
